# SIRT1 coordinates with the CRL4B complex to regulate pancreatic cancer stem cells to promote tumorigenesis

**DOI:** 10.1038/s41418-021-00821-z

**Published:** 2021-06-23

**Authors:** Shuai Leng, Wei Huang, Yang Chen, Yang Yang, Dandan Feng, Wei Liu, Tianyang Gao, Yanli Ren, Miaomiao Huo, Jingyao Zhang, Yunkai Yang, Yan Wang

**Affiliations:** 1 Key Laboratory of Immune Microenvironment and Disease (Ministry of Education), 2011 Collaborative Innovation Center of Tianjin for Medical Epigenetics, Department of Biochemistry and Molecular Biology, School of Basic Medical Sciences, Tianjin Medical University, Tianjin, 300070 China; 2 Beijing Key Laboratory of Cancer Invasion and Metastasis Research, Department of Biochemistry and Molecular Biology, School of Basic Medical Sciences, Capital Medical University, Beijing, 100069 China; 3 State Key Laboratory of Molecular Oncology, Key Laboratory of Cancer and Microbiome, National Cancer Center/National Clinical Research Center for Cancer/Cancer Hospital, Chinese Academy of Medical Sciences and Peking Union Medical College, Beijing, 100021 China

**Keywords:** Cancer stem cells, Metastasis

## Abstract

Pancreatic cancer is a common malignant tumor with poor prognosis. Recently, cancer stem cells (CSCs) were identified in several solid tumors, including pancreatic cancer. Although accumulating evidence indicates that sirtuin 1 (SIRT1) exerts biological functions in various cancers, how it contributes to tumorigenesis and metastasis of pancreatic cancer, as well as its role in CSCs, is still poorly defined. Here we show that SIRT1 interacts with the Cullin 4B (CUL4B)-Ring E3 ligase (CRL4B) complex, which is responsible for H2AK119 monoubiquitination (H2AK119ub1), collaborating as a functional unit. Genome-wide analysis of SIRT1/CUL4B targets identified a cohort of genes, including GRHL3 and FOXO3, critically involved in cell differentiation, growth, and migration. Furthermore, we found that SIRT1 and CUL4B collectively promote the proliferation, autophagy, and invasion of pancreatic cancer cells. Remarkably, we demonstrate that SIRT1/CUL4B promotes CSC-like properties, including increased stemness marker expression and sphere formation. In vivo experiments implied that SIRT1 promoted established tumor xenograft growth, increased tumor-initiating capacity in NOD/SCID mice, and increased CSC frequency. Strikingly, SIRT1 and CUL4B expression is markedly upregulated in a variety of human cancers, including pancreatic cancer. Our data provide a molecular basis for the functional interplay between histone deacetylation and ubiquitination. The results also implicate the SIRT1/CRL4B complex in pancreatic cancer metastasis and stem cell properties, thus supporting SIRT1 as a promising potential target for cancer therapy development.

## Introduction

Sirtuins are NAD^+^ dependent class III histone deacetylase enzymes with lysine deacetylation, ADP-ribosylation, and/or deacylation activities [1]; they are involved in a diverse range of cellular processes, thus governing both cancer initiation and progression [2]. SIRT1 plays an important role in tumorigenesis, development, and drug resistance by blocking aging and apoptosis, also promoting cell growth and angiogenesis [3]. It has been reported that SIRT1 inhibits apoptosis and senescence and supports the viability, proliferation, and invasion of pancreatic cancer cells [4–6]. High SIRT1 levels are associated with poorly differentiated pancreatic ductal carcinomas and poor disease outcomes [7]. Moreover, SIRT1 facilitates chemoresistance of pancreatic cancer cells by regulating adaptive responses to chemotherapy-induced stress, and combination therapy with SIRT1 inhibitor and gemcitabine was shown to have enhanced efficacy for pancreatic carcinoma [8, 9]. Despite the increasing evidence pointing to a critical role for SIRT1 in pancreatic cancer pathogenesis, the detailed mechanisms remain to be established, particularly in the development of pancreatic cancer stem cells (CSCs).

Cullin (CUL) 4-Ring E3 ligases (CRL4), with CUL4, DDB1, and ROC1 as core components, are involved in a variety of physiologically and developmentally controlled processes [10]. In mammals, there are two Cullin 4 members, CUL4A and CUL4B. The CUL4B-Ring E3 ligase (CRL4B) complex regulates transcription repression through histone H2AK119 monoubiquitination [11]. In addition, CRL4B physically associates with polycomb repressive complex 2 (PRC2) or the SUV39H1/HP1/DNMT3A complex to repress transcription of several tumor suppressors, thus promoting tumorigenesis [11, 12].

CSCs have been identified in several solid tumors, including pancreatic cancer, and are thought to exist as a distinct population, maintaining tumor cell group vitality via self-renewal and differentiation, and causing tumor metastasis, recurrence, and resistance to treatment [13, 14]. Over time, CD133^+^ cells [15] and CD44^+^ CD24^+^ EpCAM^+^ cells [14], identified as pancreatic CSC biomarkers, were shown to be enriched in pancreatic CSCs. In addition, a clear link between CSCs and the epithelial-mesenchymal transition (EMT) has been found in solid tumors [16], suggesting that similar EMT-based strategies may identify novel agents inhibiting pancreatic CSCs. Moreover, autophagy has been implicated in the homeostatic control and maintenance of stem cell self-renewal capacity [17], with blockade of autophagy reportedly reducing pancreatic CSC activity [18].

In this study, we analyzed the potential role of SIRT1 in pancreatic cancer development. Here, we reported that SIRT1 is physically associated with CRL4B complex and promotes pancreatic cancer cell proliferation, invasion, and autophagy. We demonstrated that SIRT1 and CUL4B positively regulate CSC-like features in pancreatic cancer cells. Our data indicated that SIRT1 is essential for pancreatic cancer tumorigenesis and maintenance of stemness, supporting the pursuit of SIRT1 as a target for cancer therapeutic strategies.

## Materials and methods

### Antibodies and reagents

Antibodies and their respective sources were as follows: anti-FLAG (F1408), anti-CUL4B (C9995), anti-β-actin (A1978), anti-CUL4A (C0371), anti-HDAC1 (H3284), anti-HDAC2 (H3159), anti-RbAp46/48 (R3779), anti-Vimentin (V6630), and anti-LC3B (L7543) from Sigma-Aldrich; anti-DDB1 (sc-25367), anti-MTA1 (sc-10813), and anti-MBD3 (sc-271521) from Santa Cruz Biotechnology; anti-SIRT2 (ab211033), anti-ROC1 (ab2977), anti-MTA2 (ab50209), anti-H3 (ab1791), anti-H3K14ac (ab52946), anti-OCT4 (ab19857), anti-SOX2 (ab59776), anti-c-Myc (ab32072), anti-NANOG (ab109250), and anti-H2A (ab18255) from Abcam; anti-SIRT3 (5490), anti-SIRT5 (8782), anti-SIRT6 (12486), anti-SIRT7 (5360), anti-H3K9ac (9649), anti-FOXO3 (2497), anti-p62 (88588), anti-KLF4 (12173), anti-RING1A (13069), anti-RING1B (5694), and anti-BMI1 (6964) from Cell Signaling Technology; anti-SIRT1 (07-131), anti-EED (17-10034), anti-H4K16ac (07-329), anti-H2AK119ub1 (05-678), anti-GRHL3 (ABD68), and anti-H4 (04-858) from Millipore; anti-SIRT4 (66543-1-Ig) from Proteintech; anti-CD133 (566593), anti-α-catenin (610193), anti-γ-catenin (610253), and anti-N-cadherin (610920) from BD Bioscience; anti-SAP30 (A303-551A) from Bethyl; anti-PRRX1 (YT3874) from ImmunoWay. Protein A/G Sepharose CL-4B beads were sourced from Amersham Biosciences; protease inhibitor mixture cocktail from Roche Applied Science; small interfering RNAs (siRNAs) and bafilomycin A1 from Sigma-Aldrich; short hairpin RNAs (shRNAs) from GenePharma Co., Ltd. (Shanghai, China).

### Cell culture and transfection

Cell lines used in this study were obtained from the American Type Culture Collection. PANC-1 cells and BxPC-3 cells were maintained in Dulbecco’s modified Eagle’s medium (DMEM) with 10% fetal bovine serum (FBS), AsPC-1 cells in RPMI-1640 with 10% FBS, and MIA PaCa-2 cells in DMEM containing 10% FBS, 2.5% heat-inactivated horse serum, and 1% sodium pyruvate 100 mM solution. All cells were incubated in a humidified incubator at 5% CO_2_ and 37 °C. Transfections were performed using Lipofectamine 2000 or Lipofectamine^®^ RNAiMAX Reagent (Invitrogen, Carlsbad, CA), according to the manufacturer’s instructions. Each experiment was performed in triplicate and repeated at least thrice. For RNAi experiments, at least three independent siRNA sequences were tested for each gene; the one with the highest efficiency was used. Details on the siRNA sequences covered in this article are available in Supplementary Table S1.

### Flow cytometry

Cells were resuspended in sorting buffer (1× phosphate-buffered saline (PBS); 3% FBS [v/v]; 3 mM EDTA [v/v]) before analysis. To identify pancreatic CSCs, the anti-CD133-PE antibody, or an appropriately isotype-matched control antibody, were used. Samples were analyzed using a FACSVerse (BD) flow cytometer; data were analyzed using FlowJo 9.2 software.

### Real-time quantitative PCR

Total RNA was isolated from samples using Trizol reagents (Invitrogen). Any potential DNA contamination was removed using RNase-free DNase treatment (Promega). cDNA was prepared using MMLV reverse transcriptase (Fermentas). Relative quantitation of all transcripts was detected via real-time RT-PCR performed using a Power SYBR Green PCR master mix on an ABI PRISM 7500 fast sequence detection system (Applied Biosystems, Foster City, CA). Relative quantitation of all transcripts was calculated using the comparative Ct method, with glyceraldehyde 3-phosphate dehydrogenase (GAPDH) as the internal control. This assay was performed in triplicate. All primer sequences used are listed in Supplementary Table S2.

### Immunopurification and mass spectrometry

PANC-1 cells were transfected with FLAG-tagged SIRT1 for 48 h, obtaining a cell line stably expressing FLAG-SIRT1. Anti-FLAG immunoaffinity columns were prepared using an anti-FLAG M2 affinity gel (Sigma-Aldrich), according to the manufacturer’s instructions. FLAG peptide (0.2 mg/ml; Sigma-Aldrich) was applied to the column to elute the FLAG protein complex. Fractions of the bed volume were collected and resolved on SDS-polyacrylamide gels, silver stained, and subjected to liquid chromatography-tandem mass spectrometry sequencing and data analysis.

### Immunoprecipitation (IP) and western blotting

For IP assays, cells were washed twice with cold PBS, and extracts prepared by incubating cells in lysis buffer (50 mM Tris–HCl, pH 7.4, 150 mM NaCl, 1 mM EDTA, 0.5% NP-40, 0.25% sodium deoxycholate, and a protease inhibitor cocktail) for 30 min at 4 °C; then centrifuging at 12,000 × *g* for 10 min. Next, 500 μg of cellular extract were incubated with appropriate primary antibodies or normal rabbit/mouse IgG at 4 °C overnight with constant rotation; then mixed with glutathione-sepharose beads for 2 h at 4 °C. After washing the beads four times with cell lysis buffer, captured immune complexes were subjected to SDS-PAGE, followed by IB with secondary antibodies. Immunodetection was performed using enhanced chemiluminescence (ECL System, Thermo Scientific) according to the manufacturer’s instructions. Image J software was used to quantify the protein expression.

### Fast protein liquid chromatography (FPLC)

PANC-1 cells nuclear extracts were prepared and dialyzed against buffer D (20 mM HEPES, pH 8.0, 10% glycerol, 0.1 mM EDTA, 300 mM NaCl) (Applygen Technologies, Beijing, China). Approximately 6 mg of nuclear protein was concentrated to 1 ml using Millipore Ultrafree centrifugal filter apparatus (10 kDa nominal molecular mass limit), and then applied to an 850 × 20 mm Superose 6 size exclusion column (Amersham Biosciences, Salt Lake City, UT, USA) that had been equilibrated with buffer D containing 1 mM dithiothreitol and calibrated with protein standards (blue dextran, 2000 kDa; thyroglobulin, 669 kDa; Ferritin, 440 kDa; Aldolase, 158 kDa; Ovalbumin, 43 kDa; all from Amersham Biosciences). The column was eluted at a flow rate of 0.5 ml/min and fractions were collected.

### Glutathione S-transferase (GST) pull-down experiments

GST fusion constructs were expressed in *Escherichia coli* BL21 cells, and crude bacterial lysates were prepared via sonication in cold PBS in the presence of a protease inhibitor mixture. In vitro transcription and translation experiments were performed with rabbit reticulocyte lysate (TNT Systems; Promega). In GST pull-down assays, ∼10 μg of the appropriate GST fusion proteins were mixed with 5−8 μl of in vitro-transcribed/translated products and incubated in binding buffer (0.8% bovine serum albumin in PBS in the presence of a protease inhibitor mixture) at room temperature for 30 min. The binding reaction was then added to 30 μl of Glutathione Sepharose 4B beads (GE Healthcare) and mixed at 4 °C for 2 h. Beads were then washed five times with washing buffer, resuspended in 30 μl of 2× SDS-PAGE loading buffer, and resolved on 10% gels. Protein bands were detected with specific antibodies using western blot.

### Lentivirus production and infection

Recombinant lentivirus expressing shSCR (control scrambled shRNA), shSIRT1, and shCUL4B were constructed according to the instructions from Shanghai GenePharma. Concentrated viruses were used to infect 5 × 10^5^ cells in a 60-mm dish with 8 µg/ml polybrene. Infected cells were then subjected to a selection of target expressions. All shRNA sequences are listed in Supplementary Table S3.

### Acid extraction of histones

Histones were extracted with 0.2 N HCl. Briefly, cells were harvested and washed with cold PBS containing sodium butyrate. Next, cells were resuspended in Triton extraction buffer (PBS supplemented with 0.5% Triton X-100 (v/v), 2 mM phenylmethanesulfonyl fluoride, and 0.02% NaN3(v/v)) and dissolved in ice for 10 min before centrifugation at 4°C. Cells were then washed again with Triton extraction buffer, centrifuged, and the precipitate resuspended in 0.2 N HCl; the acid was then extracted overnight at 4 °C. Next, the sample was centrifuged at 4 °C for 10 min, and the supernatant containing the extracted histone was removed and stored at −80 °C.

### Chromatin immunoprecipitation (ChIP), Re-ChIP, quantitative ChIP (qChIP), and ChIP-based deep sequencing (ChIP-seq) assays

ChIPs and Re-ChIPs were performed in PANC-1 cells as previously described [19]. Briefly, 1 × 10^7^ cells were cross-linked with 1% formaldehyde, sonicated, pre-cleared, and incubated with 2–3 µg of antibody per reaction. Complexes were washed with low and high salt buffers, and the DNA extracted and precipitated. qChIPs were performed using the TransStart Top Green qPCR SuperMix (TransGen Biotech, Shanghai, China). For Re-ChIP assays, immune complexes were eluted from the beads with 20 mM dithiothreitol. Eluents were then diluted 30-fold with ChIP dilution buffer and subjected to a second IP reaction. The final elution step was performed using a 1% SDS solution in Tris-EDTA buffer, pH 8.0. DNA template enrichment was analyzed via conventional PCR using primers specific to each target gene promoter. For ChIP-seq, a quantified 10 ng of DNA was resolved using an Agilent Technologies 2100 Bioanalyzer, with 50–250 bp fractions extracted and subjected to end-repair and 3ʹ-adenylation. Adapter-ligated libraries were amplified, purified, and selected using an Agencourt AMPure XP-Medium kit; the final library was composed of single-stranded circular DNA. In-depth whole-genome DNA sequencing was performed by the CapitalBio Corporation (Beijing, China). Sequencing data acquired from the Illumina analysis pipeline were compared with unmasked human reference genome hg19 (UCSC GRCh37) using ELAND (Illumina, San Diego, CA, USA). Peaks were called using Model-based Analysis of ChIP-Seq (MACS), following input filtering. ChIPseeker was used to analyze the genomic distribution of SIRT1- or CUL4B-binding sites. All primers used are listed in Supplementary Table S4.

### Immunofluorescence and confocal imaging

For LC3 fluorescence analysis, PANC-1 cells were infected with either EGFP-LC3 or mCherry-GFP-LC3 plasmids (Addgen). To visualize acidic lysosome compartments, cells were stained with LysoTracker Red DND-99 (Thermo Fisher Scientific). Samples were examined using an epifluorescent microscope (Olympus BX61, Tokyo, Japan). For confocal microscopy, cells seeded on coverslips were fixed in 4% formaldehyde for 10 min, and then washed with PBS thrice. Coverslips were mounted on glass slides using Vectashield with 4′, 6-diamidino-2-phenylindole.

### Wound-healing assay

PANC-1 cells in DMEM containing 10% FBS were seeded in six-well plates (Becton Dickinson) and grown to confluence; wounds were made using sterile pipette tips (200 μl, Axygen). Cells were washed with PBS and incubated in a fresh medium without FBS. Cells were imaged after 36 h of incubation at 37 °C. Assays were performed at least thrice.

### Cell invasion assay

Transwell chamber filters (Becton Dickinson) were coated with Matrigel. Next, cells were suspended in serum-free media and seeded into the upper chamber at a density of 5 × 10^4^ cells in a volume of 500 ml. Cells were then cultured in a well containing 500 ml of media with 10% FBS at 37 °C for 18–24 h. Cells on the upper side of the membrane were removed using cotton swabs, while those on the other side were stained and counted. Four high-powered fields were counted for each membrane.

### Sphere culture

A total of 5000 cells were plated in six-well ultra-low attachment plates in DMEM/F12 medium (Hyclone) without serum supplemented with B27 (50×, Invitrogen), 0.4% bovine serum albumin, 20 ng/ml bFGF, 10 ng/ml EGF, and 5 µg/ml insulin (Invitrogen). Fresh aliquots of stem cell medium were added every other day. Spheres were observed on day 5, after which they increased in size and cell number until day 15.

### Mouse xenograft models

For the tumor initiation study, PANC-1 cells transfected with stable expression of firefly luciferase (Xenogen Corporation) were infected with lentivirus carrying an empty vector or a SIRT1 expression construct. Matrigel (BD Biosciences) and these cells were injected subcutaneously into the groin of 6-week-old female NOD/SCID mice under limiting dilutions 1 × 10^6^, 1 × 10^5^, 1 × 10^4^, 1 × 10^3^, 5 × 10^2^, 2 × 10^2^, or 50 cells. Seven mice were tested in each group. For bioluminescence imaging, mice were injected intraperitoneally with 200 mg/g D-luciferin in PBS. Ten minutes after the injection, mice were anesthetized, and bioluminescence imaged using a charge-coupled device camera (IVIS; Xenogen). Bioluminescence images were obtained in a 15-cm field-of-view, binning (resolution) factor of 8, 1/f stop, open filter, and an imaging time of 30 s to 2 min. Bioluminescence from relative optical intensity was defined manually. Photon flux was normalized to the background, which was defined based on the relative optical intensity drawn from a mouse not injected with luciferin. Animal handling and procedures were approved by Tianjin Medical University Institutional Animal Care Center.

### Tissue specimens and immunohistochemistry

Immediately after surgical removal, samples were frozen in liquid nitrogen and maintained at –80 °C until analysis. Samples were fixed in 4% paraformaldehyde (Sigma-Aldrich) at 4 °C overnight and then processed, paraffin-embedded, sectioned, and stained with hematoxylin and eosin according to a standard protocol. For immunohistochemistry staining, 8-μm-thick sample sections were incubated overnight in a humidification chamber at 4 °C, followed by a 2 h incubation with horseradish peroxidase-bound secondary antibodies. Staining was completed via incubation with diaminobenzidine (DAB) substrate for 5–10 min, resulting in a brown precipitate at the antigen site.

### Statistical analysis

Results are reported as the means ± SD unless otherwise noted. Comparisons were performed using a two-tailed unpaired *t* test. SPSS V.17.0 was used for statistical analysis. Tumor data sets were downloaded from http://www.ncbi.nlm.nih.gov/geo, with each GSE number shown in the figures.

## Results

### Systematic profiling of sirtuin effects on stem-like phenotypes in pancreatic cancer cells

**Fig. 1:**
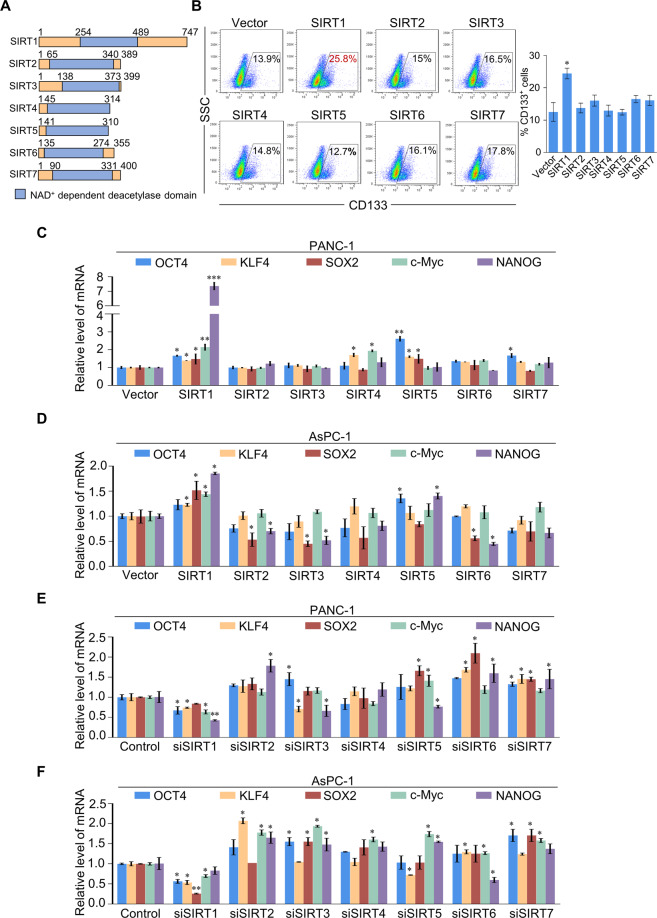
Systematic profiling of sirtuin effects on pancreatic cancer stem cells. **A** Domain architectures of human sirtuins. NAD^+^ dependent deacetylase domains are colored in blue. **B** CD133 staining of PANC-1 cells was assessed using flow cytometry. The box shows the percentage of CD133^+^ cells in detected PANC-1 cells. **C**, **D** RT-qPCR data for the relative mRNA expression levels of OCT4, KLF4, SOX2, c-Myc, and NANOG in SIRT1-7 overexpressing PANC-1 and AsPC-1 cells. **E**, **F** RT-qPCR data for the relative mRNA expression levels of OCT4, KLF4, SOX2, c-Myc, and NANOG in SIRT1-7 knockdown PANC-1 and AsPC-1 cells. **B**–**F** Error bars represent the mean ± SD of three independent experiments. ∗*p* < 0.05, ∗∗*p* < 0.01, ∗∗∗*p* < 0.001; two-tailed unpaired *t* test.

The seven human sirtuin members (SIRT1–7) share a conserved NAD^+^ dependent deacetylase domain (Fig. 1A). To investigated whether sirtuins affect stem-like phenotypes in pancreatic cancer cells, FLAG-tagged SIRT1–7 were stably expressed in PANC-1 cells; using flow cytometry, CD133^+^ cell content increased in cells overexpressing SIRT1 (Fig. 1B); there was no significant change in other cells. Stem cell markers were upregulated in PANC-1 and AsPC-1 cells overexpressing SIRT1, while overexpression of other sirtuin family members only altered the expression of some CSC markers (Fig. 1C and D). Western blotting further verified the plasmids used in these experiments (Fig. S1A and B). Accordingly, expression of these factors declined in response to SIRT1 knockdown. Moreover, knocking down SIRT2–7 did not cause a unified change in the expression of these CSC markers (Fig. 1E and F). Meanwhile, we validated the knockdown efficiency of small interfering RNA (siRNA) targeted to each of SIRT1–7 mRNA (Fig. S1C and D). In addition, SIRT1 had little effect on the expression of other SIRTs in PANC-1 cells (Fig. S1E). Flow cytometry to sort CD133^−^ and CD133^+^ PANC-1 cells and real-time quantitative PCR (RT-qPCR) and western blotting showed that the expression of SIRT1 was upregulated in CD133^+^ cells (Fig. S1F). Taken together, these results suggest that SIRT1 appears to be linked to CSC-associated properties, such as CD133 expression and stemness gene levels.

### SIRT1 is physically associated with the CRL4B complex

**Fig. 2:**
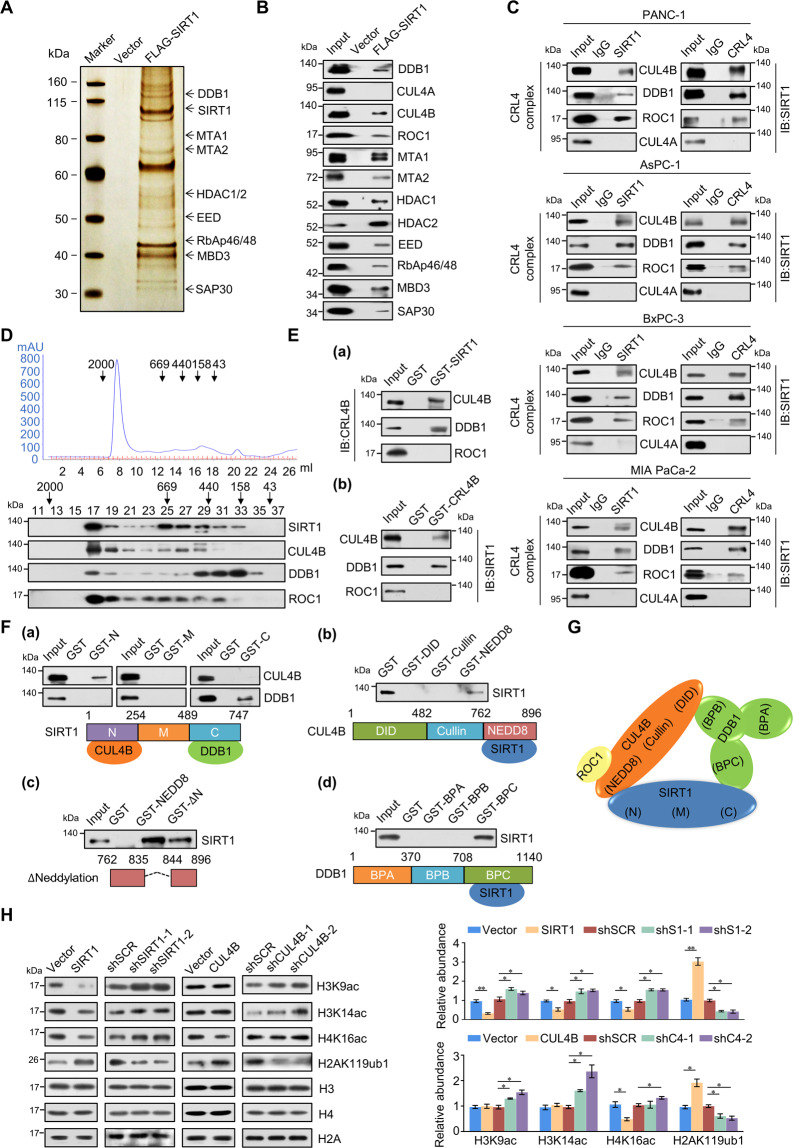
SIRT1 is physically associated with the CRL4B complex. **A** Immunoaffinity purification and mass spectrometry analysis of SIRT1-containing protein complexes. Whole-cell extracts from PANC-1 cells stably expressing FLAG (Vector) or FLAG-SIRT1 were immunopurified using anti-FLAG affinity columns and eluents with FLAG peptide. Eluates were resolved using SDS-PAGE and silver-stained. Protein bands were retrieved and analyzed using mass spectrometry. **B** Western blot analysis of the purified fractions using antibodies against the indicated proteins. **C** Co-IP assays in PANC-1, AsPC-1, BxPC-3, and MIA PaCa-2 cells with anti-SIRT1, followed by IB with antibodies against the indicated proteins, or with antibodies against the indicated proteins followed by IB with anti-SIRT1. **D** SIRT1 and CRL4B complex co-fractionation using fast protein liquid chromatography. PANC-1 cell nuclear extracts were first fractionated on a DEAE sepharose column, and then on a Superose 6 gel filtration column. Fractions were analyzed using western blotting. Molecular weight standards (kDa) are shown at the top. **E** GST pull-down assays with bacterially expressed GST-fused proteins and in vitro-transcribed/translated proteins. **F** Identification of the essential domains required for interaction. (a) GST pull-down assays with GST-fused SIRT1 N-terminal domain (N), NAD^+^- dependent deacetylase catalytic core domain (M), or amino C-terminal domain (C) and in vitro-transcribed/translated CUL4B or DDB1. (b) GST pull-down assays with GST-fused CUL4B DDB1-interacting domain (DID), Cullin domain (Cullin), or NEDD8 neddylation domain (NEDD8) and in vitro-transcribed/translated SIRT1. (c) GST pull-down assays with GST-fused CUL4B NEDD8 neddylation domain (NEDD8) or NEDD8 domain with neddylation site deletion (∆Neddylation) and in vitro-transcribed/translated SIRT1. ∆N, ∆Neddylation. (d) GST pull-down assays with three GST-fused DDB1 propeller domains (BPA, BPB, or BPC) *and* in vitro-transcribed/translated SIRT1. **G** Schematic diagram depicting molecular interactions between SIRT1 and CRL4B. **H** Western blotting analysis of H3K9ac, H3K14ac, H4K16ac, and H2AK119ub1 global levels in PANC-1 cells upon FLAG-tagged SIRT1 or CUL4B overexpression or knockdown. Histone H3, H4, or H2A served as the loading control. Quantitative protein expression by gray scanning. S1, SIRT1; C4, CUL4B. Error bars represent the mean ± SD of three independent experiments. ∗*p* < 0.05, ∗∗*p* < 0.01; two-tailed unpaired *t* test.

To better understand the mechanistic role of SIRT1 in pancreatic cancer, affinity purification and mass spectrometry analysis were emplyed and the results showed that SIRT1 was co-purified with various epigenetic factors (Fig. 2A). Among the listed proteins, the association of SIRT1 with MTA1 [20], HDAC1/2 [21, 22], EED [23], and SAP30 [24] has been previously reported. Detailed results of mass spectrometric analysis are provided in Supplementary Table S5. The presence of these proteins in the SIRT1-associated complex was further confirmed using western blot (Fig. 2B). Besides the proteins previously reported to interact with SIRT1, the newly identified SIRT1-associated protein, DDB1, indicated that SIRT1 may physically associate with CUL4B and ROC1, components of the CRL4B complex, rather than CUL4A, a constituent of the CRL4A complex (Fig. 2B). To further confirm the in vitro interaction between SIRT1 and the CRL4B complex, we performed co-IP assays in four pancreatic carcinoma cell lines, and the results demonstrated that SIRT1 co-immunoprecipitated with the CRL4B complex, rather than CRL4A complex (Fig. 2C). To ascertain the existence of a complex composed of SIRT1/CRL4B, protein fractionation experiments were performed with nuclear proteins using FPLC. Western blotting showed that the elution pattern of SIRT1 largely overlapped with that of CRL4B components (Fig. 2D), supporting the argument that SIRT1 and the CRL4B complex may cooperate functionally in vivo.

Next, the results of GST pull-down assays indicated that SIRT1 interacted directly with CUL4B and DDB1 (Fig. 2E). Moreover, the N-terminal of SIRT1 as responsible for CUL4B binding, while the C-terminal was necessary for DDB1 binding (Fig. 2F(a)). Results also indicated that the CUL4B NEDD8 domains involvement in directly interacting with SIRT1 (Fig. 2F(b)). CUL4B neddylation sites have been previously reported at amino acids 836–843 [25, 26]. The results showed that the CUL4B NEDD8 domain with neddylation site deletion still interacted directly with SIRT1 (Fig. 2F(c)), indicating that the interaction between SIRT1 and CUL4B independent on neddylation. DDB1 consists of three propeller (BP) domains: BPA, BPB, and BPC, where the BPB domain is responsible for the CUL4-DDB1 interaction [27, 28]. GST pull-down assays demonstrated that the DDB1 BPC domain was responsible for the interaction with SIRT1 (Fig. 2F(d)). GST-fused proteins purified from BL21 *Escherichia coli* are shown in Figure S2A–S2D. These results not only further support the specific interaction between SIRT1 and the CRL4B complex, but also revealed the molecular mechanism involved in the formation of the SIRT1/CRL4B complex (Fig. 2G).

To further explore the functional relationship between SIRT1 and CUL4B, we investigated whether SIRT1 or CUL4B would alter the global levels of H3K9ac, H3K14ac, H4K16ac, and H2AK119ub1. Western blot indicating that SIRT1-overexpression decreased H3K9ac, H3K14ac, and H4K16ac, while drastically increasing H2AK119ub1 (Fig. 2H). Accordingly, knockdown of SIRT1 in PANC-1 cells, these histone sites exhibited the opposite trend. CUL4B-overexpression markedly increased H2AK119ub1 and decreased H4K16ac; in response to CUL4B knockdown, H2AK119ub1 markedly decreased, while H3K9ac, H3K14ac, and H4K16ac slightly increased (Fig. 2H). Western blotting further verified the plasmids and shRNAs used in these experiments (Fig. S2E). PRC1 also catalyzes histone H2AK119 monoubiquitylation [29], however, IP assays demonstrated that SIRT1 did not interact with the PRC1 complex in PANC-1 cells (Fig. S2F). These results provide evidence supporting the specific mechanisms underpinning the interaction between SIRT1 and the CRL4B complex; thus identifying the functional connectedness of SIRT1 and CUL4B.

### Genome-wide identification of SIRT1/CRL4B complex transcription targets

**Fig. 3:**
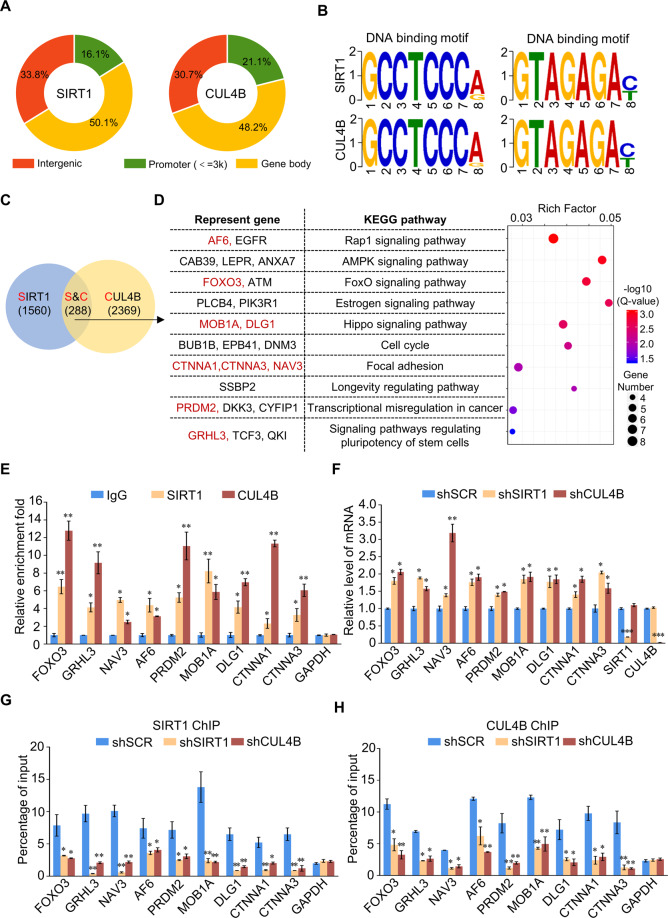
Genome-wide identification of SIRT1/CRL4B complex transcription targets. **A** Genomic distribution of SIRT1 and CUL4B determined using ChIP-seq analysis. **B** SIRT1- and CUL4B-bound motifs analyzed using the MEME suite. **C** Venn diagram of overlapping promoters bound by SIRT1 and CUL4B in PANC-1 cells. Numbers represent the number of promoters targeted by the indicated proteins. **D** A bubble chart of the 10 enriched KEGG pathways comprising the 288 overlapping target genes of SIRT1 and CUL4B. Representative genes of each pathway are also shown. The Rich Factor represents the ratio of the number of target genes to the total genes annotated in a pathway. A greater Rich Factor indicates greater intensity. The *Q*-value represents the corrected *p*-value, ranging from 0~1; a lower *Q*-value indicates greater intensity. **E** Verification of ChIP-seq results using qChIP analysis of indicated genes in PANC-1 cells. Results are represented as fold change over control, with GAPDH as a negative control. **F** PANC-1 cells were infected with lentiviruses carrying the indicated shRNAs. RT-qPCR data for the relative mRNA expression levels of the indicated genes. **G**, **H** PANC-1 cells were infected with lentivirus carrying the indicated shRNA. qChIP analysis of selected promoters was performed using antibodies against SIRT1 (**G**) or CUL4B (**H**). Results are presented as percentage of input, with GAPDH as a negative control. **E**–**H** Error bars represent the mean ± SD of three independent experiments. ∗*p* < 0.05, ∗∗*p* < 0.01; two-tailed unpaired *t* test.

We next used ChIP-seq to analyze genome-wide SIRT1/CRL4B complex transcriptional targets. We found 10455 and 12591 SIRT1- and CUL4B-specific binding peaks, respectively (Fig. 3A). Moreover, we found that SIRT1 and CUL4B had similar binding motifs (Fig. 3B), supporting the notion that they physically interact and are functionally linked. Next, data from SIRT1 (1560 genes) and CUL4B (2369 genes) were analyzed for overlapping DNA promoter sequences; these promoters represented co-targets of the SIRT1/CRL4B complex (Fig. 3C). We identified a total of 288 unique promoters targeted by SIRT1 and CUL4B, which were then classified into various cellular signaling pathways using the Kyoto Encyclopedia of Genes and Genomes (KEGG) pathway software (Fig. 3D), including Rap1, AMPK, FoxO, Hippo, cell cycle, focal adhesion, and regulating pluripotency of stem cells that are critically involved in tumor initiation and progression.

qChIP analysis showed that SIRT1 and CUL4B strong enrichment on the promoters of selected genes involved in classical pathways, including FOXO3, GRHL3, NAV3, AF6, PRDM2, MOB1A, DLG1, CTNNA1, and CTNNA3, all implicated in tumor suppression (Fig. 3E). RT-qPCR further showed that transcription levels of the target genes partially increased in PANC-1 cells upon SIRT1 or CUL4B knockdown (Fig. 3F). Moreover, we studied the effect of SIRT1 depletion on CUL4B recruitment in endogenous target loci, and vice versa. ChIP experiments showed that SIRT1 and CUL4B recruitment to their target promoters was reduced in both SIRT1- or CUL4B-depleted PANC-1 cells (Fig. 3G and H). Therefore, SIRT1 and CRL4B may mutually promote each other’s recruitment and/or stabilization on target promoters, forming a transcriptional repression complex that inhibits the expression of target genes.

### Regulation of FOXO3 and GRHL3 via the SIRT1/CRL4B complex

**Fig. 4:**
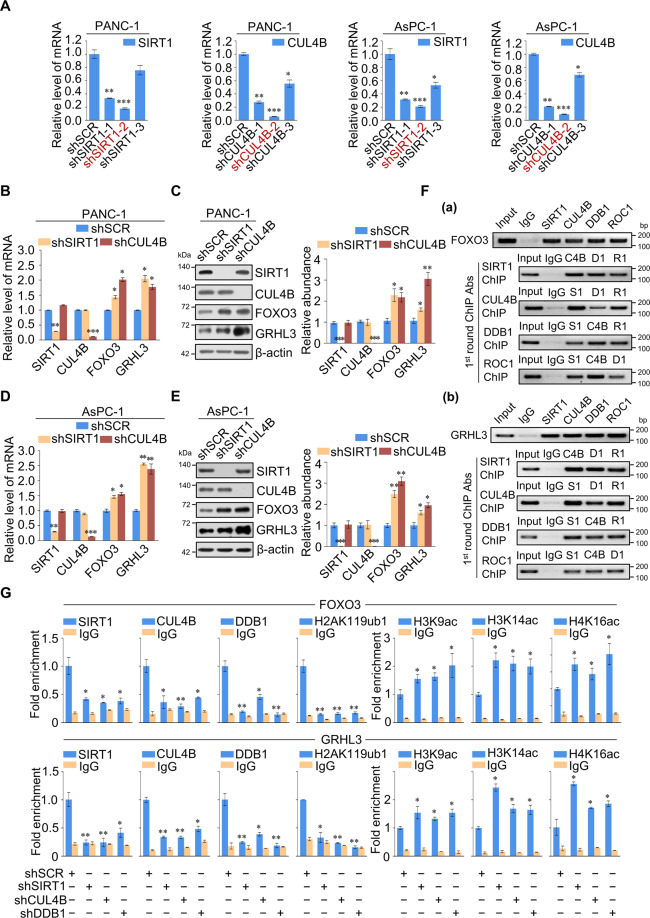
Tumor suppressor genes FOXO3 and GRHL3 are cotargeted by the SIRT1/CRL4B complex. **A** Efficiency of shRNA targeting either SIRT1 or CUL4B. PANC-1 and AsPC-1 cells were infected with lentivirus carrying control shRNA (shSCR) or shRNA targeting either SIRT1 or CUL4B. Knockdown efficiencies of SIRT1 and CUL4B were verified using RT-qPCR. We chose shSIRT1-2 and shCUL4B-2 (marked in red) for further study. **B**, **C** Clones in which SIRT1 or CUL4B were stably knocked down were compared with the parental cell lines to evaluate the levels of FOXO3 and GRHL3 mRNA (**B**) and protein (**C**) in PANC-1 cells. mRNA levels were normalized to those of GAPDH; β-actin served as a loading control for western blotting. Protein expression was quantified by gray scanning. **D**, **E** Clones in which SIRT1 or CUL4B were stably knocked down were compared with the parental cell lines to evaluate the levels of FOXO3 and GRHL3 mRNA (**D**) and protein (**E**) in AsPC-1 cells. mRNA levels were normalized to those of GAPDH; β-actin served as a loading control for western blotting. Protein expression was quantified by gray scanning. **F** SIRT1 and the CRL4B complex were found in the same protein complex on FOXO3 and GRHL3 promoters. ChIP and Re-ChIP experiments were performed in PANC-1 cells with the indicated antibodies. **G** qChIP analysis of the recruitment of indicated proteins on FOXO3 and GRHL3 promoters in PANC-1 cells after transfection with control shRNA (shSCR) or shRNAs targeting SIRT1, DDB1, or CUL4B. Purified rabbit IgG was used as a negative control. **A**–**E**, **G** Error bars represent the mean ± SD of three independent experiments. ∗*p* < 0.05, ∗∗*p* < 0.01, ∗∗∗*p* < 0.001; two-tailed unpaired *t* test.

Next, we assessed the lentivirus-delivered shRNA package targeting SIRT1 and CUL4B mRNA (Fig. 4A), selecting the most effective package (marked in red) for the following experiments. FOXO3 is a well-established tumor suppressor gene involved in various cellular processes [30]; GRHL3 is necessary for differentiation and has a tumor-suppressing role [31–33]. We therefore investigated the transcriptional regulation of FOXO3 and GRHL3 by the SIRT1/CRL4B complex. SIRT1 or CUL4B knockdown resulted in increased expression of FOXO3 and GRHL3 in PANC-1 (Fig. 4B and C) and AsPC-1 (Fig. 4D and E) cells. SIRT1/CRL4B complex-mediated regulation of FOXO3 and GRHL3 was further investigated using ChIP or ChIP/Re-ChIP experiments in PANC-1 cells (Fig. 4F). These results support that SIRT1 and the CRL4B complex occupy FOXO3 and GRHL3 promoters as one protein complex. In addition, qChIP analyses showed that knockdown of SIRT1, CUL4B, or DDB1 expression resulted in a significant reduction in the recruitment of corresponding proteins to FOXO3 and GRHL3 promoters (Fig. 4G), implying that SIRT1, CUL4B, and DDB1 act as a whole, each component essential for the complex to bind to chromatin. Notably, SIRT1 knockdown not only resulted in increased H3K9ac, H3K14ac, and H4K16ac at the FOXO3 and GRHL3 promoters, but also significantly decreased H2AK119ub1 levels; CUL4B or DDB1 knockdown led to similar results, suggesting that the SIRT1/CRL4B complex binds to FOXO3 and GRHL3 promoters as a whole, catalyzing the ubiquitination and deacetylation of histones (Fig. 4G). This further confirms that SIRT1 and the CRL4B complex are functionally associated through the transcriptional repression of a cohort of target genes, such as FOXO3 and GRHL3.

### SIRT1 and CUL4B collectively promote the proliferation, autophagy, and metastasis of pancreatic cancer cells

**Fig. 5:**
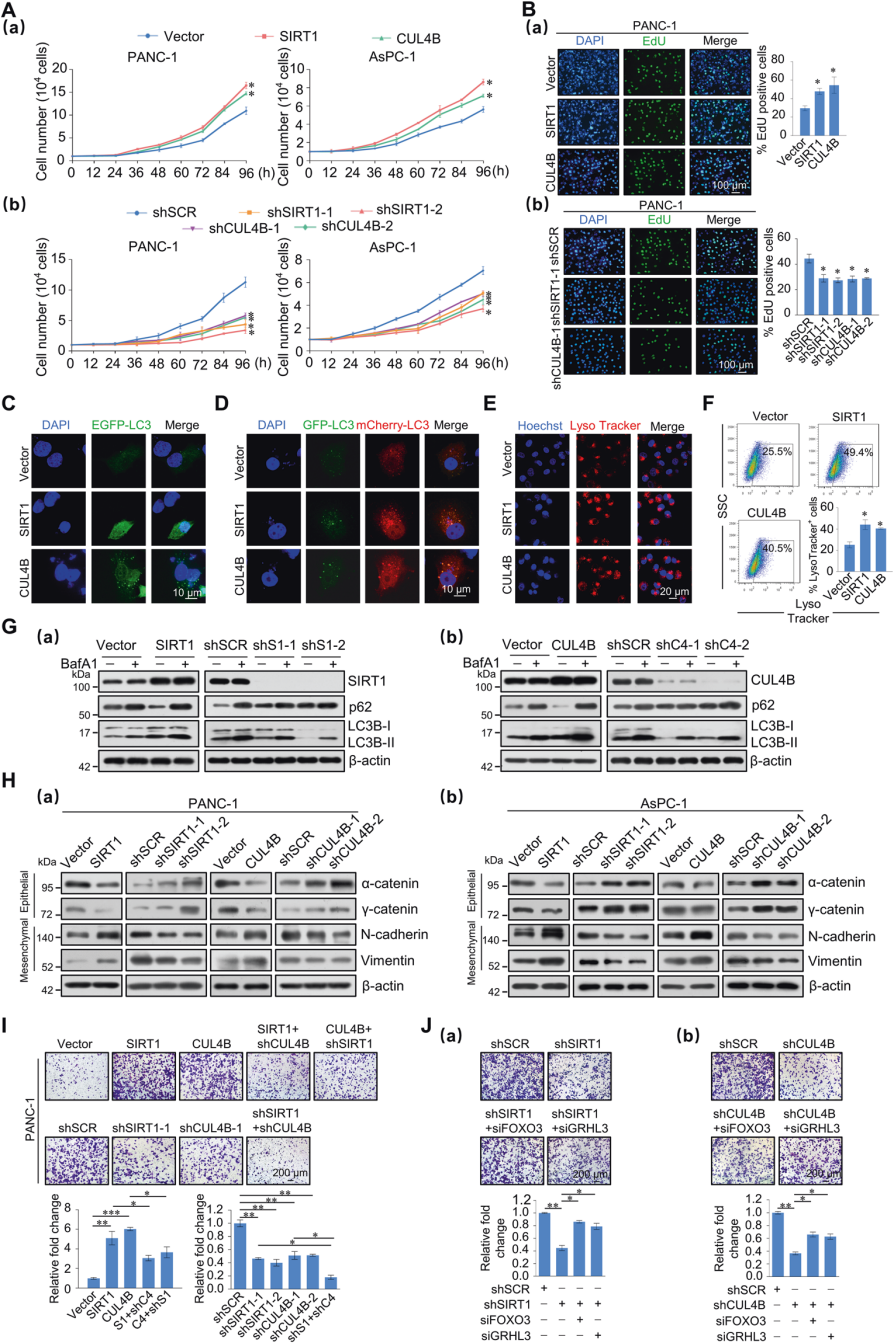
SIRT1 and CUL4B promote pancreatic cancer cell proliferation, autophagy, and invasion in vitro. **A** SIRT1 and CUL4B promote cellular proliferation. Growth curve analysis was performed on PANC-1 and AsPC-1 cells transfected with vector, SIRT1, CUL4B or shSCR, two different shRNA against SIRT1 or CUL4B. **B** PANC-1 cells were incubated with EdU for 2 h. A fluorescence microscope was used to detect EdU. **C** Representative fluorescence images of PANC-1 cells transiently expressing GFP-LC3B, with SIRT1 or CUL4B stably overexpressed. **D** Representative fluorescence images of PANC-1 cells transiently expressing mCherry-GFP-LC3, with SIRT1 or CUL4B stably overexpressed. **E** LysoTracker Red-stained PANC-1 cells stably overexpressing SIRT1 or CUL4B exhibited an expanded lysosomal area. **F** LysoTracker Red-stained PANC-1 cells stably overexpressing SIRT1 or CUL4B were assessed by flow cytometry. **G** Expression of p62 and LC3B was measured by western blotting in PANC-1 cells with stable SIRT1 or CUL4B overexpression or depletion. Bafilomycin A1 (BafA1; 200 nM, 2 h). β-actin served as a loading control. S1, SIRT1; C4, CUL4B. **H** Expression of indicated epithelial or mesenchymal markers was measured by western blotting in PANC-1 and AsPC-1 cells with SIRT1 or CUL4B overexpression or depletion. β-actin served as a loading control. S1, SIRT1; C4, CUL4B. **I** Transwell invasion assays of PANC-1 cells following stable transfection with corresponding virus. Invading cells were stained and counted. Images represent one field under microscopy in each group. S1, SIRT1; C4, CUL4B. **J** Transwell invasion assays were performed in PANC-1 cells infected with lentivirus carrying shSIRT1 or shCUL4B, or in combination with siFOXO3 or siGRHL3. Invading cells were stained and counted. Images represent one field under microscopy in each group. **A**–**B**, **F**, **I**–**J** Error bars represent the mean ± SD of three independent experiments. ∗*p* < 0.05, ∗∗*p* < 0.01, ∗∗∗*p* < 0.001; two-tailed unpaired *t* test.

We next investigated the role of the SIRT1/CRL4B complex in proliferation, autophagy, and metastasis of pancreatic cancer cells. Growth curve analysis showed that SIRT1 and CUL4B promote cell proliferation (Fig. 5A). Next, 5-ethynyl-2′-deoxyuridine (EdU) results revealed that SIRT1 or CUL4B overexpression associated with a marked percentage increase in EdU-labeled cells, while SIRT1 or CUL4B knockdown cells showed a much lower percentage of these cells (Fig. 5B and Fig. S3A). This indicates that the SIRT1/CRL4B complex promotes the proliferation of pancreatic cells in vitro.

In pancreatic cancer cells, high levels of autophagy have been observed under basal conditions [34]. Moreover, it has been reported that autophagy blockade reduces pancreatic CSC activity [18]. Therefore, we next investigated the role of SIRT1 and CUL4B in autophagy. SIRT1 or CUL4B overexpression in PANC-1 and AsPC-1 cells increased processing of LC3B-I to LC3B-II, and reduced p62 accumulation as shown via western blotting (Fig. S3B). Similarly, when SIRT1 or CUL4B knockdown, these proteins exhibited the opposite trend. We next stably transfected EGFP-LC3 or tandem-tagged mCherry-GFP-LC3 plasmids into PANC-1 cells overexpressing SIRT1 or CUL4B, to monitor the subcellular localization of LC3. In EGFP-LC3 PANC-1 cells, SIRT1 or CUL4B overexpression increased LC3 puncta (Fig. 5C). In mCherry-GFP-LC3 PANC-1 cells, SIRT1 or CUL4B overexpression revealed an increase in GFP^-^/mCherry^+^ (red puncta) autolysosomes and, to a lesser extent, GFP^+^/mCherry^+^ (yellow puncta) phagophores/autophagosomes (Fig. 5D). Next, staining of lysosomal compartments with LysoTracker Red exhibited an expanded lysosomal area in PANC-1 cells overexpressing SIRT1 or CUL4B (Fig. 5E). In addition, flow cytometry showed that the LysoTracker^+^ cell content increased in cells overexpressing SIRT1 or CUL4B (Fig. 5F). We performed autophagic flux analysis using bafilomycin A1 (BafA1), an inhibitor of autophagosomal and lysosomal fusion. After BafA1 treatment, the increase in SIRT1 and CUL4B was associated with LC3-II accumulation. Defects in autophagic flux caused by knockdown of SIRT1 and CUL4B were also confirmed by western blot analysis (Fig. 5G and Fig. S3C). These results indicate that not only autophagic flux, but also lysosomal function, are enhanced via SIRT1 or CUL4B overexpression.

The impact of SIRT1 or CUL4B on migration potential was investigated using a wound-healing assay and the results showed that SIRT1 and CUL4B promoted PANC-1 cells migration rates (Fig. S3D). Next, western blots showed that epithelial marker expression, such as α- and γ-catenin, decreased, while mesenchymal markers, including N-cadherin and Vimentin, increased upon SIRT1 or CUL4B overexpression (Fig. 5H and Fig. S3E). With individual knockdown of SIRT1 or CUL4B in PANC-1 and AsPC-1 cells, these EMT markers exhibited the opposite trend. In addition, SIRT1 has been reported to deacetylate and stabilize the EMT-inducer PRRX1 [35]. Our results showed PRRX1 expression increased in SIRT1- or CUL4B-overexpressing PANC-1 and AsPC-1 cells and decreased in response to SIRT1 or CUL4B knockdown (Fig. S3F). The western blotting results shown in Figure S3F verify the SIRT1 and CUL4B overexpression and knockdown efficiency in these experiments. Moreover, results from transwell invasion assays in PANC-1 and AsPC-1 cells showed that SIRT1 or CUL4B overexpression resulted in a greater than twofold increase in cell invasion, while knockdown of SIRT1 or CUL4B resulted in apparent decreases in cell invasion potential (Fig. 5I and Fig. S3G). The effect of SIRT1 or CUL4B overexpression diminished with CUL4B or SIRT1 knockdown, and with co-knockdown of SIRT1 and CUL4B, cell invasion ability significantly weakened (Fig. 5I and Fig. S3G). Therefore, SIRT1 and CUL4B are functionally interdependent during invasion promotion. Furthermore, we designed siRNA targeted to either FOXO3 or GRHL3 mRNA (Fig. S3H). SIRT1 or CUL4B knockdown in PANC-1 cells decreased cell invasion potential, which was partially rescued via the co-knockdown of FOXO3 or GRHL3, indicating that the SIRT1/CRL4B complex could promote pancreatic cancer invasion through repression of FOXO3 and GRHL3 (Fig. 5J). These results indicate that the SIRT1/CRL4B complex promotes the migration and invasion potential of pancreatic cancer cells, partially by repressing FOXO3 and GRHL3.

### SIRT1 and CUL4B promote pancreatic cancer stemness

**Fig. 6:**
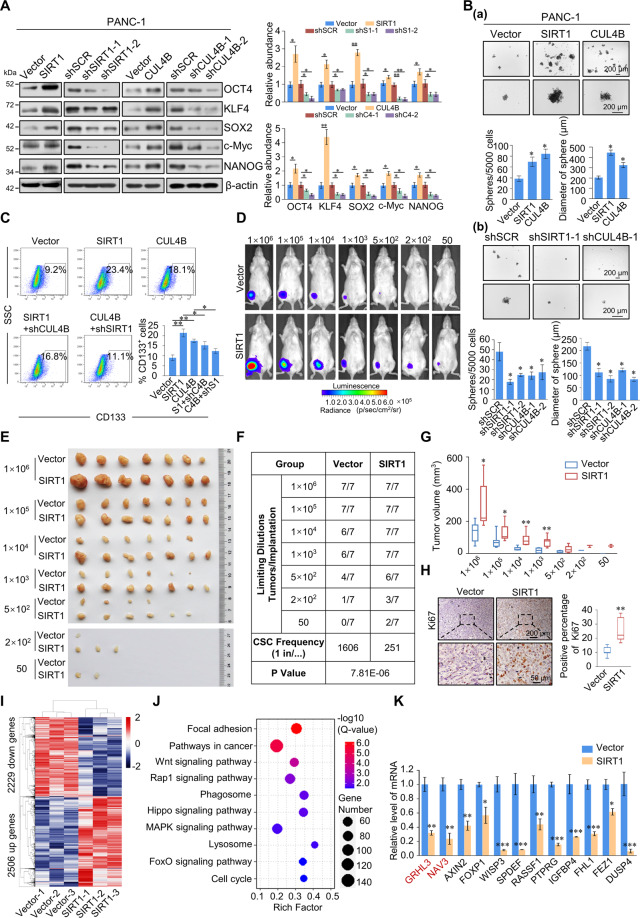
SIRT1 and CUL4B promote pancreatic cancer stem cell properties and SIRT1 promotes pancreatic tumor xenograft growth in NOD/SCID mice by promoting the development of cancer stemness. **A** Western blot analysis of stem cell marker expression in PANC-1 cells with stably overexpressed or knocked down SIRT1 and CUL4B. β-actin served as a loading control. Protein expression was quantified by gray scanning. S1, SIRT1; C4, CUL4B. **B** PANC-1 cells with stably overexpressed or knocked down SIRT1 and CUL4B. Representative images of spheres grown in suspension culture for 15 days. Cells were plated in an ultra-low attachment six-well plate (5000/well). **C** CD133 staining of PANC-1 cells was assessed using flow cytometry. The box shows the percentage of CD133^+^ cells. **D** PANC-1 cells were engineered to stably express firefly luciferase, carrying an empty vector or SIRT1. These cells were injected subcutaneously into the groin (*n* = 7) of 6-week-old female NOD/SCID mice under limiting dilutions 1 × 10^6^, 1 × 10^5^, 1 × 10^4^, 1 × 10^3^, 5 × 10^2^, 2 × 10^2^, or 50 cells. Tumors were quantified using bioluminescence imaging 4 weeks after initial implantation. Representative in vivo bioluminescent images are shown. **E** Tumor specimens were examined using in vitro measurements. **F** CSC frequency was calculated using Extreme Limiting Dilution Analysis (ELDA) software (http://bioinf.wehi.edu.au/software/elda/index.html). **G** The volume of tumor specimens was detected in vitro. The data shown are the mean ± SD. ∗*p* < 0.05, ∗∗*p* < 0.01; two-tailed unpaired *t* test. **H** Ki67 staining was performed via immunohistochemistry on fixed sections. All tumor cells were counted in at least five random fields. **I** Heatmap of differentially expressed genes (fold-change > 1.2, *p* < 0.001) in control (Vector-1, Vector-2, and Vector-3) and SIRT1-overexpressing (SIRT1-1, SIRT1-2, and SIRT1-3) tumor samples. (Blue) downregulated genes; (red) upregulated genes. **J** Ten enriched KEGG pathways comprised of upregulated or downregulated genes mediated by SIRT1 overexpression. The Rich Factor represents the ratio of differentially expressed genes to the total genes annotated in a pathway. A greater Rich Factor indicates greater intensity. The *Q*-value represents the corrected p-value ranging 0–1; a lower *Q*-value indicates greater intensity. **K** Total RNA was extracted from tumor samples, and RT-qPCR analysis of mRNAs performed in control vector or SIRT1-overexpressing. **A**–**C**, **H**, **K** Error bars represent the mean ± SD of three independent experiments. ∗*p* < 0.05, ∗∗*p* < 0.01, ∗∗∗*p* < 0.001; two-tailed unpaired *t* test.

Next, we investigated whether SIRT1 and CUL4B affect stem-like phenotypes in pancreatic cancer cells. Stem cell markers all increased in PANC-1 and AsPC-1 cells stably expressing SIRT1 or CUL4B (Fig. 6A and Fig. S4A). Furthermore, the expression of these factors decreased in response to SIRT1 or CUL4B knockdown. The overexpression and knockdown efficiency of SIRT1 and CUL4B were verified using western blotting (Fig. S4B). To further elucidate whether SIRT1 and CUL4B promote the development of pancreatic cancer cells into CSCs, repopulating from single cells, we analyzed the effect of SIRT1 and CUL4B on sphere formation. The number and size of spheres increased in PANC-1 and AsPC-1 cells stably expressing SIRT1 or CUL4B, and decreased in response to knockdown SIRT1 or CUL4B (Fig. 6B and Fig. S4C). Next, flow cytometry showed that the number of CD133^+^ cells increased after SIRT1 and CUL4B overexpression, an effect partially rescued by CUL4B and SIRT1 knockdown (Fig. 6C). These results indicate that SIRT1/CRL4B complex promotes pancreatic CSC properties.

To further confirm that SIRT1 targets the pancreatic CSC population in vivo, we established mouse xenograft models. The growth of implanted cells was visualized via bioluminescence 4 weeks after injection (Fig. 6D and Fig. S4D). Cells stably expressing SIRT1 had markedly increased tumor-initiating capacity, with no tumors observed after the introduction of 50 cells from the control vector group (Fig. 6E). These functional assays allowed us to calculate the frequency of tumor-initiating cells. The SIRT1-overexpressing group showed a significant increase in CSC frequency compared to the control group (Fig. 6F). Furthermore, SIRT1 significantly promoted the growth of pancreatic tumors (Fig. 6G). This demonstrates that SIRT1 dramatically induces stemness in pancreatic cancer cells, thus increasing the growth of established tumor xenografts.

Consistent with accelerated tumor growth, the proportion of Ki67-positive cells was significantly higher in the SIRT1-overexpressing group compared with the control vector group (Fig. 6H). We further extracted total protein and RNA from tumor samples and verified SIRT1 overexpression by western blotting (Fig. S4E). Moreover, we investigated genome-wide effects of SIRT1-overexpression using high-throughput RNA deep sequencing (RNA-seq). Compared to the control, we identified a total of 2506 upregulated genes and 2229 downregulated genes (fold change > 1.2, *p* < 0.001) in SIRT1-overexpressing tumor samples (Fig. 6I). Using KEGG database, the results of pathway enrichment analyses revealed that these differentially expressed genes are not only involved in focal adhesion, Wnt, Hippo, cell cycle, and other pathways closely related to tumor growth and stemness, but also enriched in phagosome and lysosomal pathways related to autophagy (Fig. 6J). Next, we selected 12 known tumor suppressor genes implicated in cancer development, including GRHL3, NAV3, AXIN2, FOXP1, WISP3, SPDEF, RASSF1, PTPRG, IGFBP4, FHL1, FEZ1, and DUSP4, and using RT-qPCR, validated that their expression decreased in SIRT1-overexpressing tumor samples (Fig. 6K), thus further validating our RNA-seq results. As our studies have shown that the SIRT1/CRL4B complex transcriptionally inhibits GRHL3, we hypothesized that SIRT1 inhibits the differentiation of cancer cells by inhibiting GRHL3 expression, thus promoting the stemness and tumorigenesis of pancreatic cancer. To further delineate the molecular pathways that depend on SIRT1, RNA-seq analysis was preformed using PANC-1 cells. Compared to the control, cells with siSIRT1 showed 3651 upregulated genes and 2211 downregulated genes (fold-change > 1.2, *p* < 0.001) (Fig. S4F). These target genes are not only involved in tumorigenesis and stemness-related pathways, but also enriched in lysosomal pathway related to autophagy (Fig. S4G and H). These results suggest that SIRT1 is involved in regulating various pathways closely related to tumor growth, autophagy, and stemness, as well as promoting pancreatic CSC development.

### Expression of SIRT1 and CUL4B is upregulated in multiple carcinomas and is a potential cancer biomarker

**Fig. 7:**
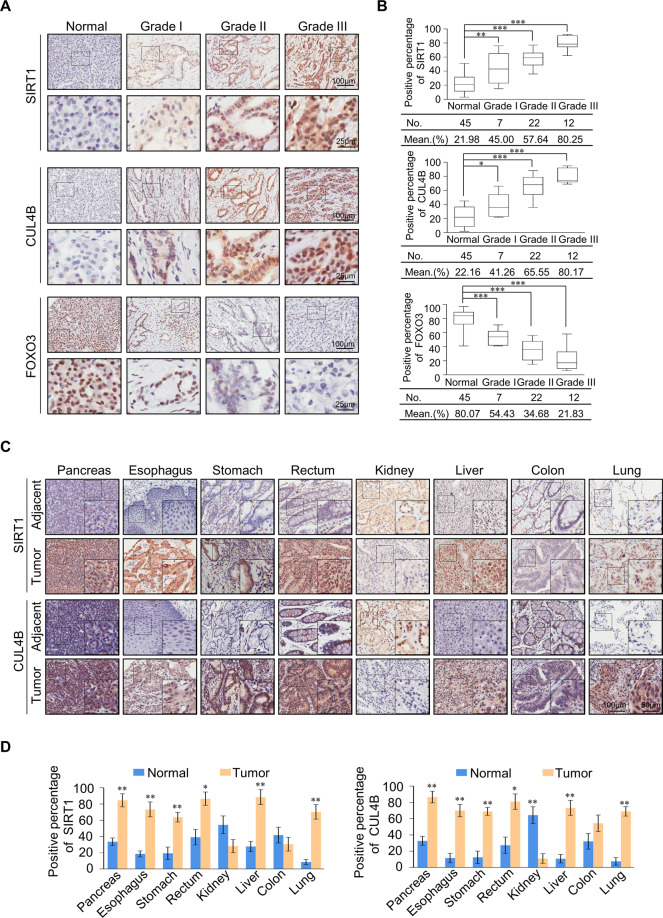
SIRT1 is a potential cancer biomarker. **A** Immunohistochemical staining of SIRT1, CUL4B, and FOXO3 in normal pancreatic tissue and pancreatic tumors (histological grades I, II, & III). **B** Positively stained nuclei (in percentages) in grouped samples were analyzed using a two-tailed unpaired *t*-test. ∗*p* < 0.05, ∗∗*p* < 0.01, ∗∗∗*p* < 0.001. **C**, **D** SIRT1 and CUL4B are upregulated in multiple carcinomas. Immunohistochemical staining of SIRT1 and CUL4B in paired tumor tissues versus adjacent normal tissues are shown. Representative images of 200-fold magnifications of each type of paired tumor section are presented. Error bars represent the mean ± SD. ∗*p* < 0.05, ∗∗*p* < 0.01; two-tailed unpaired *t* test.

We collected 86 pancreatic carcinoma samples from pancreatic cancer patients and performed tissue microarrays via immunohistochemical staining, to examine the expression of SIRT1, CUL4B, and FOXO3 (Fig. 7A). SIRT1 and CUL4B were found to be significantly upregulated in tumors, with their level of expression positively correlated with tumor histological grades, while FOXO3 expression was negatively correlated with tumor histological grades (Fig. 7B). Furthermore, analysis of a published clinical dataset (GSE15471) revealed that compared with normal pancreatic tissues, SIRT1 and CUL4B expression increased in pancreatic tumor samples, while FOXO3 and GRHL3 significantly decreased (Fig. S5A). To investigate whether the effect of SIRT1 and CUL4B could be extended to a broader scope of cancers, we collected several carcinoma samples on which we performed tissue microarrays and immunohistochemical staining to examine SIRT1 and CUL4B expression (Fig. 7C). The results indicated that, in addition to pancreatic cancer, SIRT1 and CUL4B are also significantly upregulated in esophagus, stomach, rectum, liver, and lung carcinomas, compared with adjacent normal tissues (Fig. 7D). Next, we used Gene Expression Profiling Interactive Analysis to analyze SIRT1 and CUL4B expression profiles in The Cancer Genome Atlas tumor samples and corresponding normal tissues. The results showed a significant positive correlation between SIRT1 and CUL4B expression and lymphoid neoplasm diffuse large B-cell lymphoma, brain lower grade glioma, pancreatic adenocarcinoma, and thymoma (Fig. S5B). These analyses show that SIRT1 and CUL4B have similar expression trends in a variety of cancers, further supporting the idea that the SIRT1/CRL4B complex plays key roles in cancer as an organic whole. In summary, our analyses show that SIRT1 and CUL4B are upregulated in multiple carcinomas and are potential cancer biomarkers.

## Discussion

**Fig. 8:**
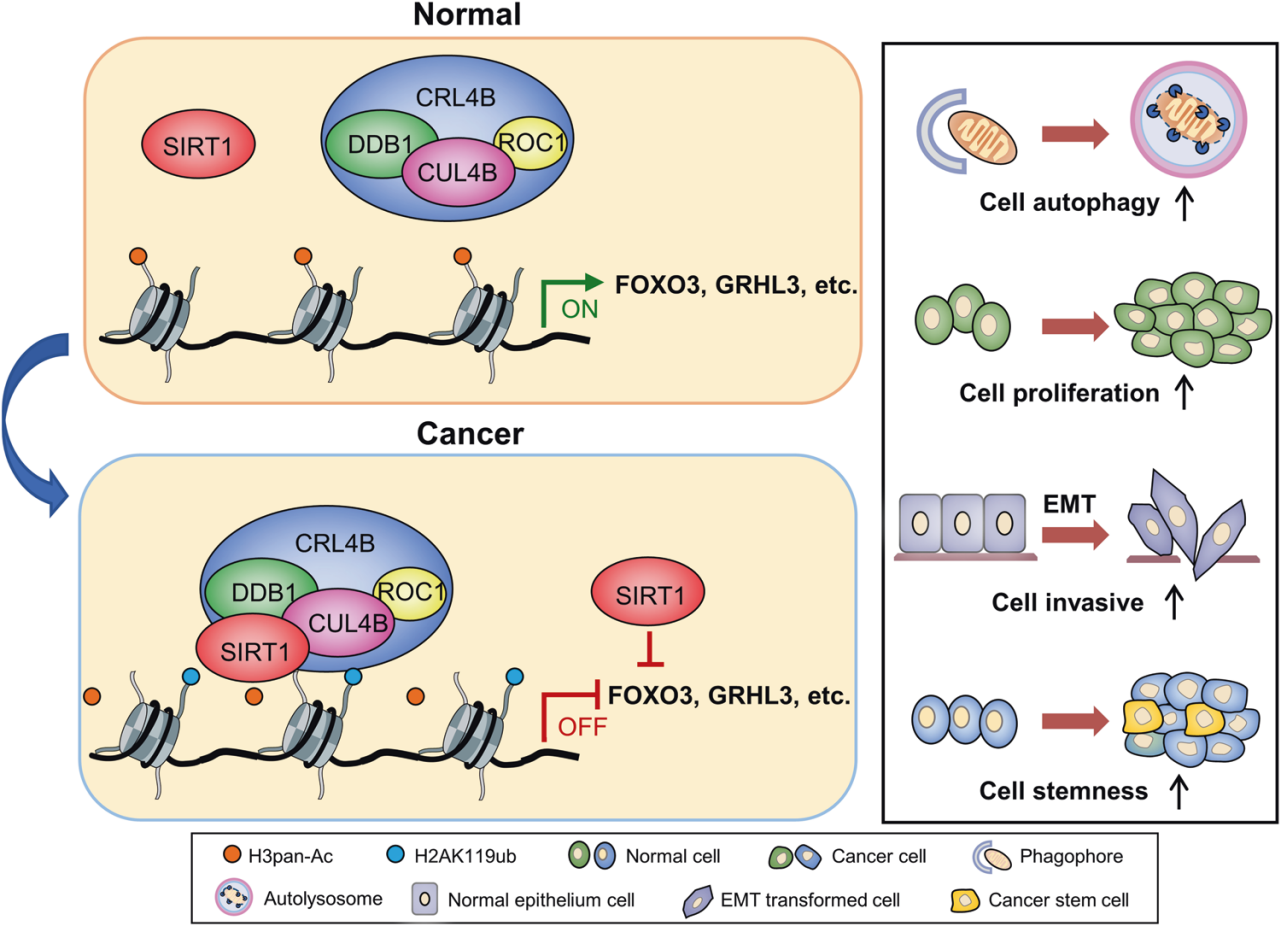
Graphic model as discussed in the text. The proposed regulatory mechanisms of the SIRT1/CRL4B complex in controlling proliferation, autophagy, invasion, and stemness of pancreatic carcinogenesis.

In this study, we demonstrated that SIRT1 cooperates with the CRL4B complex in transcriptional inhibition, also participating in various biological processes associated with pancreatic cancer, including proliferation, autophagy, invasion, and stemness. The proposed regulatory mechanisms of the SIRT1/CRL4B complex in controlling EMT and stem cell properties of pancreatic carcinogenesis is described in Fig. 8.

SIRT1 upregulation has been observed in pancreatic cancers and is associated with worse overall survival rates in patients with pancreatic cancer [6, 7, 36]. Previous studies have shown an important pro-tumoral role for SIRT1 in pancreatic cancer. SIRT1 regulates acinar-to-ductal metaplasia by deacetylating pancreatic transcription factor-1a and β-catenin [5]. E-cadherin transcription inhibition is directly related to SIRT1 in pancreatic cancer. In fact, SIRT1 forms a protein complex that can silence E-cadherin promoter by interacting with twist and MBD1 [37]. These findings provide insight into the mechanistic function of SIRT1 as an oncogene. In contrast, limited studies indicated that SIRT1 reduced cell proliferation and tumor formation in pancreatic cancer models [38, 39]. Therefore, SIRT1 may regulate the delicate balance between the suppression and promotion of tumorigenesis according to its activity level, spatiotemporal distribution, tumorigenesis stage, and tumor microenvironment [40]. In this study, we further confirmed that SIRT1 can promote mesenchymal marker expression, downregulate epithelial marker expression, and promote pancreatic cancer cell invasion, thus emphasizing its positive role in the induction of EMT. Notably, in some experimental models of cancer, forced induction of EMT in epithelial tumor cells substantially increases their ability to initiate tumors [41].

The NOD/SCID mouse xenograft models showed that SIRT1 not only significantly promoted tumor growth, but also tumor-initiating capacity and CSC frequency, thus clarifying its role in promoting pancreatic CSCs. Furthermore, we identified GRHL3 as the target gene of the SIRT1/CRL4B complex. Interestingly, that expression of GRHL3 significantly reduced in tumor tissues overexpressing SIRT1. Previous studies have shown that GRHL3 is necessary for differentiation [32]. SIRT1/CRL4B complex transcriptionally inhibited GRHL3 expression, thereby inhibiting cell differentiation, thus providing an important molecular basis for SIRT1 role in pancreatic CSCs.

Autophagy is involved in controlling and maintaining the self-regulation ability of stem cells [17], while CSC pluripotency requires autophagy homeostasis [42]. In addition, blocking autophagy can reduce pancreatic CSCs activity and potentiate the tumoricidal effects of chemotherapeutic drugs. SIRT1 can directly deacetylate LC3 [43], Atg5, Atg7, and Atg8 [44], each of which are important components of the autophagy mechanism, thus promoting autophagy in the starvation state. In addition, SIRT1 deacetylated H4K16 activated by the AMPK cascade during starvation, leading to BRD4 translocation of the ATG gene promoter, thus activating autophagy [45], indicating the role of SIRT1 epigenetic modification in autophagy. Although CUL4B regulates autophagy via the JNK signal in diffuse large B-cell lymphoma [46], its role in autophagy has not been reported in other cancers and studies. Our findings indicated that the SIRT1/CRL4B complex functions as a whole, while co-phenotypic experiments with SIRT1 showed that CUL4B positively correlated with autophagy in pancreatic cancer. The specific mechanism and the relationship between autophagy and stemness require further investigation.

We found that SIRT1 and CRL4B interact and cooperate as a functional unit, catalyzing the ubiquitination and deacetylation of histones, and thus inhibiting the transcription of target genes. Furthermore, the deacetylation of SIRT1 modified H3K9, H3K14, and H4K16, and H2AK119 monoubiquitination cooperated to expand the SIRT1 enzymatic library to ubiquitin activity. Similarly, the enzymatic repertoire of CUL4B was extended to deacetylation activity. In addition, we demonstrated that SIRT1 and CUL4B are enriched in the promoters of target genes as FOXO3 and GRHL3. After either SIRT1 or CUL4B knockdown, their enrichment was greatly reduced, supporting the hypothesis that SIRT1 and CUL4B are a complex. Based on these findings, we speculate that in the absence of SIRT1, the CRL4B complex at promoters would not be stably tethered, thus increasing acetylation and reducing H2AK119 mono-ubiquitination. The SIRT1/CRL4B complex formed on targeted promoters, producing a deacetylated H3K9, H3K14, and H4K16/H2AK119ub1 co-repressed ‘histone code’, thus transcriptionally inhibiting the target genes. This newly identified cooperation in histone modification provides new clues into the functional interaction between different enzyme activities and the mechanisms behind epigenetic transcription regulation.

Our study revealed that SIRT1 and CRL4B interact and cooperate as a functional unit, thereby providing a new transcription regulatory model, as well as a novel molecular basis for histone deacetylation and ubiquitination in chromatin remodeling. Furthermore, the SIRT1/CRL4B complex contributes to the epigenetic silencing of tumor suppressors, also playing an important role in pancreatic cancer tumorigenesis and regulating the properties of CSCs. Thus, SIRT1 and CUL4B are potential oncogenes and biomarkers and may serve as targets for tumor therapy.

## Supplementary information

Supplementary Figure legends

Supplementary Tables

Figure S1

Figure S2

Figure S3

Figure S4

Figure S5

## Data Availability

The accession numbers for the ChIP-seq, RNA-seq (mouse xenograft models tumor samples), and RNA-seq (siSIRT1) data reported in this paper are Gene Expression Omnibus (GEO): GSE163337, GSE163101, and GSE171118, respectively.
