# Supplementary material for: SIRT1 coordinates with the CRL4B complex to regulate pancreatic cancer stem cells to promote tumorigenesis
Source: Cell Death Differ. 2021 Jun 23;28(12):3329–43. doi: 10.1038/s41418-021-00821-z (PMC8630059; doi:10.1038/s41418-021-00821-z)
Supplement: Supplementary file 1 — Supplementary Figure legends [file 41418_2021_821_MOESM1_ESM.docx]

**Supplementary Figure Legends**

**Figure S1 (related to Figure 1). Detection of SIRT1-7 overexpression and knockdown efficiency.**

(A and B) Western blot analysis of SIRT1-7 expression in PANC-1 and AsPC-1 cells transfected with FLAG-tagged SIRT1-7 plasmids. β-actin served as a loading control.

(C and D) Efficiency of seven siRNAs targeting SIRT1-7. PANC-1 and AsPC-1 cells were infected with siRNA (control) or seven different siRNAs targeting SIRT1-7. Knockdown efficiencies of SIRT1-7 were verified via western blotting. β-actin served as a loading control.

(E) RT-qPCR data and western blotting results of the levels of SIRT1-7 mRNA and protein in SIRT1-overexpressing or SIRT1-knockdown PANC-1 cells. mRNA levels were normalized to those of GAPDH; β-actin served as a loading control for western blotting. Protein expression was quantified by gray scanning.

(F) Flow cytometry was performed to sort CD133^−^ (P3) and CD133^+^ (P2) PANC-1 cells and RT-qPCR and western blotting were conducted to evaluate the levels of SIRT1-7 mRNA and protein in CD133^−^ and CD133^+^ PANC-1 cells. mRNA levels were normalized to those of GAPDH; β-actin served as a loading control for western blotting. Protein expression was quantified by gray scanning.

(E and F) Error bars represent the mean ± SD of three independent experiments. ∗*p* < 0.05, ∗∗*p* < 0.01, ∗∗∗*p* < 0.001; two-tailed unpaired *t* test.

**Figure S2 (related to Figure 2). SIRT1 interacts with the CRL4B complex rather than the PRC1 complex.**

(A–D) GST-fused proteins purified from BL21 *Escherichia coli* used in Figure 2.

(E) Western blot analysis of SIRT1 or CUL4B expression in PANC-1 cells transfected with a different plasmid or lentivirus. β-actin served as a loading control.

(F) IP assays in PANC-1 cells using anti-SIRT1 followed by IB using antibodies against the indicated proteins.

**Figure S3 (related to Figure 5). SIRT1 and CUL4B promote a variety of malignant phenotypes of pancreatic cancer cells in vitro.**

(A) AsPC-1 cells were incubated with EdU for 2 h. A fluorescence microscope was used to detect EdU.

(B) Expression of p62 and LC3B was measured by western blotting in PANC-1 and AsPC-1 cells with stable SIRT1 or CUL4B overexpression or depletion. β-actin served as a loading control. Protein expression was quantified by gray scanning. S1, SIRT1; C4, CUL4B.

(C) Expression of p62 and LC3B was measured by western blotting in PANC-1 cells with stable SIRT1 or CUL4B overexpression or depletion. Bafilomycin A1 (BafA1; 200 nM, 2 h). β-actin served as a loading control. Protein expression was quantified by gray scanning. S1, SIRT1; C4, CUL4B.

(D) SIRT1 and CUL4B promote cellular migration. A wound-healing assay was performed on PANC-1 cells transfected with a vector, SIRT1, or CUL4B, or transfected with shSCR, two different shRNA against SIRT1 or CUL4B.

(E) Expression of indicated epithelial or mesenchymal markers was measured by western blotting in PANC-1 and AsPC-1 cells with SIRT1 or CUL4B overexpression or depletion. β-actin served as a loading control. Protein expression was quantified by gray scanning. S1, SIRT1; C4, CUL4B.

(F) Western blot analysis of SIRT1, CUL4B, and PRRX1 expression in PANC-1 and AsPC-1 cells transfected with different lentiviruses. β-actin served as a loading control.

(G) Transwell invasion assays of AsPC-1 cells following stable transfection with corresponding virus. Invading cells were stained and counted. Images represent one field under microscopy in each group.

(H) Efficiency of siRNA targeting either FOXO3 or GRHL3. PANC-1 cells were infected with siRNA (control) or siRNA targeting either FOXO3 or GRHL3. Knockdown efficiencies were verified using RT-qPCR. siFOXO3-3 and siGRHL3-3 (marked in red) were chosen for further study.

(A–D, E, G–H) Error bars represent the mean ± SD of three independent experiments. ∗*p* < 0.05, ∗∗*p* < 0.01, ∗∗∗*p* < 0.001; two-tailed unpaired *t* test.

**Figure S4 (related to Figure 6). SIRT1 and CUL4B promote pancreatic cancer stemness.**

(A) Western blot analysis of stem cell marker expression in AsPC-1 cells with stably overexpressed or knocked down SIRT1 and CUL4B. β-actin served as a loading control. Protein expression was quantified by gray scanning. S1, SIRT1; C4, CUL4B.

(B) Western blot analysis of SIRT1 or CUL4B expression in PANC-1 and AsPC-1 cells transfected with different lentiviruses in Figure 3 and Figure S4. β-actin served as a loading control.

(C) AsPC-1 cells with stably overexpressed or knocked down SIRT1 and CUL4B. Representative images of the indicated spheres grown in suspension culture for 15 days. These cells were plated in an ultra-low attachment 6-well plate at a density of 5,000/well.

(D) Tumors were quantified using bioluminescence imaging 4 weeks after initial implantation. Data shown are the mean ± SD. ∗∗*p* < 0.01; two-tailed unpaired *t* test.

(E) Total protein was extracted from tumor samples, and western blotting used to examine SIRT1 protein levels. β-actin served as a loading control.

(F) Heatmap of differentially expressed genes (fold-change > 1.2, *p* < 0.001) in control (Control-1 and Control-2) and SIRT1 knockdown (siSIRT1-1 and siSIRT1-2) PANC-1 cells. (Blue) down-regulated genes; (red) up-regulated genes.

(G) Ten enriched KEGG pathways comprised of upregulated or downregulated genes mediated by SIRT1 knockdown. The Rich Factor represents the ratio of differentially expressed genes to the total genes annotated in a pathway. A greater Rich Factor indicates greater intensity. The Q-value represents the corrected p-value ranging 0–1; a lower Q-value indicates greater intensity.

(H) Heatmaps of differentially expressed genes in the lysosome pathway.

(A and C) Error bars represent the mean ± SD of three independent experiments. ∗*p* < 0.05, ∗∗*p* < 0.01; two-tailed unpaired *t* test.

**Figure S5 (related to Figure 7). SIRT1 and CUL4B expression profiles in multiple carcinomas.**

(A) Analysis of public datasets (GSE15471) for the expression of FOXO3 or GRHL3 and SIRT1 or CUL4B in pancreatic carcinomas. ∗*p* *<* 0.05, ∗∗*p* < 0.01, ∗∗∗*p* < 0.001, ∗∗∗∗*p* < 0.0001; two-tailed unpaired *t* test.

(B) Dot plot of SIRT1 and CUL4B expression profiles across all tumor samples and paired normal tissues using GEPIA.
