# Supplementary material for: SIRT1 coordinates with the CRL4B complex to regulate pancreatic cancer stem cells to promote tumorigenesis
Source: Cell Death Differ. 2021 Jun 23;28(12):3329–43. doi: 10.1038/s41418-021-00821-z (PMC8630059; doi:10.1038/s41418-021-00821-z)
Supplement: Supplementary file 2 — Supplementary Tables [file 41418_2021_821_MOESM2_ESM.docx]

**Supplementary Table S1. The siRNA sequences**

| **siRNA sequences** | |
| --- | --- |
| siSIRT1 | GUGUCAUGGUUCCUUUGCA |
| siSIRT2 | CUACUCCUGCGCUGCUACA |
| siSIRT3 | CUAGCAUGUUGGUCUCCUU |
| siSIRT4 | GAGAAACUCGGAAAGCUGU |
| siSIRT5 | CAGCAUCCCAGUUGAGAAA |
| siSIRT6 | CUCACUUUGUUACUUGUUU |
| siSIRT7 | CCCUGAAGCUACAUGGGAA |
| siGRHL3-1 | CACAUCAAGUCAGGCGAGU |
| siGRHL3-2 | CUGUUACCCACCACUGAUA |
| siGRHL3-3 | GUGUUCAUCGGCGUAAACU |
| siFOXO3-1 | GAAUGAUGGGCUGACUGAA |
| siFOXO3-2 | CGAUUCAUGCGGGUCCAGA |
| siFOXO3-3 | CGAAUCAGCUGACGACAGU |

**Supplementary Table S2. The primers used in RT-qPCR**

| **Genes** | **Forward Primer Sequences** | **Reverse Primer Sequences** |
| --- | --- | --- |
| SIRT1 | TCTCTGTCACAAATTCATAGCC | CAAAGGAACCATGACACTGAA |
| OCT4 | ATCACCCTGGGATATACACAG | CTGCTTTGCATATCTCCTGA |
| KLF4 | CCCACATGAAGCGACTTCCC | CAGGTCCAGGAGATCGTTGAA |
| SOX2 | GCCTGGGCGCCGAGTGGA | GGGCGAGCCGTTCATGTAGGTCTG |
| c-Myc | AAACTTGAACAGCTACGGAAC | ATTTGAGGCAGTTTACATTATGG |
| NANOG | TCTGGACACTGGCTGAATCCT | CGCTGATTAGGCTCCAACCAT |
| GRHL3 | GGACCTCACTCCCCTTGAAAG | CAGTGGTGGGTAACAGGTAGC |
| NAV3 | AGCCTGTGCATACTGCTCTTC | TGATTTTAACGCAAGCTGACAAG |
| AXIN2 | CAACACCAGGCGGAACGAA | GCCCAATAAGGAGTGTAAGGACT |
| FOXP1 | TGGCATCTCATAAACCATCAGC | GGTCCACTCATCTTCGTCTCAG |
| WISP3  SPDEF | GGGCACTGGACCATTAGATACA | TGAGTAGTCACAATACAGCCCT |
| SPDEF | CAGTGCCCGGTCATTGACA | CAGCCGGTATTGGTGCTCT |
| RASSF1 | AGGACGGTTCTTACACAGGCT | TGGGCAGGTAAAAGGAAGTGC |
| PTPRG | TGGAACCGTGTTGGTGGATTT | CAACGTAGCCTTCTGTCAACG |
| IGFBP4 | GGTGACCACCCCAACAACAG | GAATTTTGGCGAAGTGCTTCTG |
| FHL1 | TGCTGCCTGAAATGCTTTGAC | GCCAGAAGCGGTTCTTATAGTG |
| FEZ1 | CCACTGGTGAGTCTGGATGAA | CGGAAGAAAAATTCTCAAGCTCG |
| DUSP4 | GGCGGCTATGAGAGGTTTTCC | TGGTCGTGTAGTGGGGTCC |
| FOXO3 | CGGACAAACGGCTCACTCT | GGACCCGCATGAATCGACTAT |
| AF6 | CTGGACCTGTTCGAGATCAGC | CGAGCGTTTCGATTACATCTTGA |
| PRDM2 | AATCAGAACACTACTGAGCCTGT | ACCAATCCGGGTCTTGTCAAC |
| MOB1A | CAGCAGCCGCTCTTCTAAAAC | CCTCAGGCAACATAACAGCTTG |
| DLG1 | GCAGGAGGTACGGACAACC | ATTGACCCGCAATCTTCCATC |
| CTNNA1 | GGGGATAAAATTGCGAAGGAGA | GTTGCCTCGCTTCACAGAAGA |
| CTNNA3 | AAGCTACTGGAGCCTCTCATAA | CTTTGCGAACTTCCTCAAGTGA |
| CUL4B | GGCAACTGGAATAGAGGATG | TGTTCTTCAACCGTTTCTTTC |
| GAPDH | GTCAACGGATTTGGTCGTAT | GAACATGTAAACCATGTAGTTGA |

**Supplementary Table S3. The shRNA sequences**

| **shRNA sequences** | |
| --- | --- |
| shSIRT1-1 | CTAGGAATGTTGAAAGTATTG |
| shSIRT1-2 | CTAGGAATGTTGAAAGTATTG |
| shSIRT1-3 | CCATGAAGTATGACAAAGATG |
| shCUL4B-1 | GCCACGTACCGATACAGAAGA |
| shCUL4B-2 | GGATTCATTGGATAGCGTTCT |
| shCUL4B-3 | GGATAGAACTTACGTTCTTCA |
| shDDB1 | TCCACTAGATCGCGATAATAA |

**Supplementary Table S4. The primers used in qChIP**

| **Genes** | **Forward Primer Sequences** | **Reverse Primer Sequences** |
| --- | --- | --- |
| GRHL3 | GACGATCTTACTTTGGCGTGTC | TGTTTCCCTGCCTGGCTC |
| FOXO3 | GTTTCCTATGCCCTTTTA | CATGCTCAAATTAGCCAC |
| NAV3 | ATCCCTTCTCCAAGTGCC | GTGGCTTCAGTCTGTCTG |
| AF6 | CTGACCGAGCCTCCCTGT | TGGCAACAAGGAAAAGAC |
| PRDM2 | CCAGCCTAAACTTTGATA | CAACTTATTGTGGTCCCT |
| MOB1A | GATACTACCATATTGCCC | GGTGCCAAAAAGGTTAGG |
| DLG1 | TTCTTTACCTGCTAATCG | CTCGTATCTTCCCTCTGT |
| CTNNA1 | TCCTTTGTTCATTTGTAT | CAGGTGGAGATTGTTGTG |
| CTNNA3 | TGATGTTACCCTTCACTG | AAACAGCCTGACAAGCAC |
| GAPDH | AGCCACATCGCTCAGACACC | CCCATACGACTGCAAAGACCC |

**Supplementary Table S5. Mass spectrometry results of SIRT1-containing protein complex in PANC-1 cells**

| **Description** | **Score** | **Coverage** | **Proteins** | **Unique Peptides** | **Peptides** | **PSMs** | **AAs** |
| --- | --- | --- | --- | --- | --- | --- | --- |
| SIRT1 | 18034.20 | 79.25 | 5 | 27 | 73 | 6635 | 747 |
| KAP1 | 667.36 | 39.4 | 4 | 11 | 24 | 249 | 835 |
| AP1 | 326.53 | 45.02 | 2 | 9 | 10 | 104 | 331 |
| DBC1 | 163.44 | 60.49 | 5 | 1 | 7 | 57 | 162 |
| USP22 | 152.60 | 22.48 | 13 | 11 | 11 | 62 | 525 |
| MTA2 | 120.08 | 39.67 | 1 | 21 | 23 | 45 | 668 |
| HDAC2 | 117.09 | 31.76 | 17 | 6 | 13 | 51 | 488 |
| RBAP48 | 104.00 | 18.82 | 13 | 6 | 7 | 34 | 425 |
| RbAp46 | 99.64 | 18.75 | 5 | 5 | 6 | 36 | 416 |
| HDAC1 | 90.81 | 27.59 | 10 | 5 | 12 | 43 | 482 |
| P53 | 91.90 | 42.94 | 127 | 16 | 16 | 45 | 354 |
| CDK1 | 73.11 | 31.31 | 103 | 8 | 9 | 31 | 297 |
| CK2A1 | 72.27 | 34.03 | 14 | 9 | 11 | 30 | 385 |
| AROS | 66.52 | 37.50 | 2 | 6 | 6 | 19 | 136 |
| MTA1 | 61.35 | 18.63 | 18 | 9 | 11 | 25 | 703 |
| MBD3 | 55.04 | 25.43 | 9 | 7 | 7 | 20 | 291 |
| CCNB1 | 49.42 | 29.56 | 10 | 9 | 9 | 19 | 433 |
| DNMT1 | 36.15 | 6.75 | 7 | 10 | 10 | 17 | 1511 |
| CSNK2B | 29.62 | 23.72 | 6 | 4 | 4 | 12 | 215 |
| P300 | 19.83 | 2.57 | 17 | 6 | 6 | 9 | 2414 |
| EED | 12.83 | 9.70 | 6 | 3 | 3 | 5 | 361 |
| DDB1 | 9.73 | 3.27 | 5 | 3 | 3 | 5 | 1009 |
| KAT2B | 6.52 | 3.37 | 2 | 1 | 3 | 5 | 832 |
| SAP30 | 5.85 | 12.27 | 1 | 2 | 2 | 3 | 220 |
