# Supplementary figures and images for: SIRT1 coordinates with the CRL4B complex to regulate pancreatic cancer stem cells to promote tumorigenesis
Source: Cell Death Differ. 2021 Jun 23;28(12):3329–43. doi: 10.1038/s41418-021-00821-z (PMC8630059; doi:10.1038/s41418-021-00821-z)

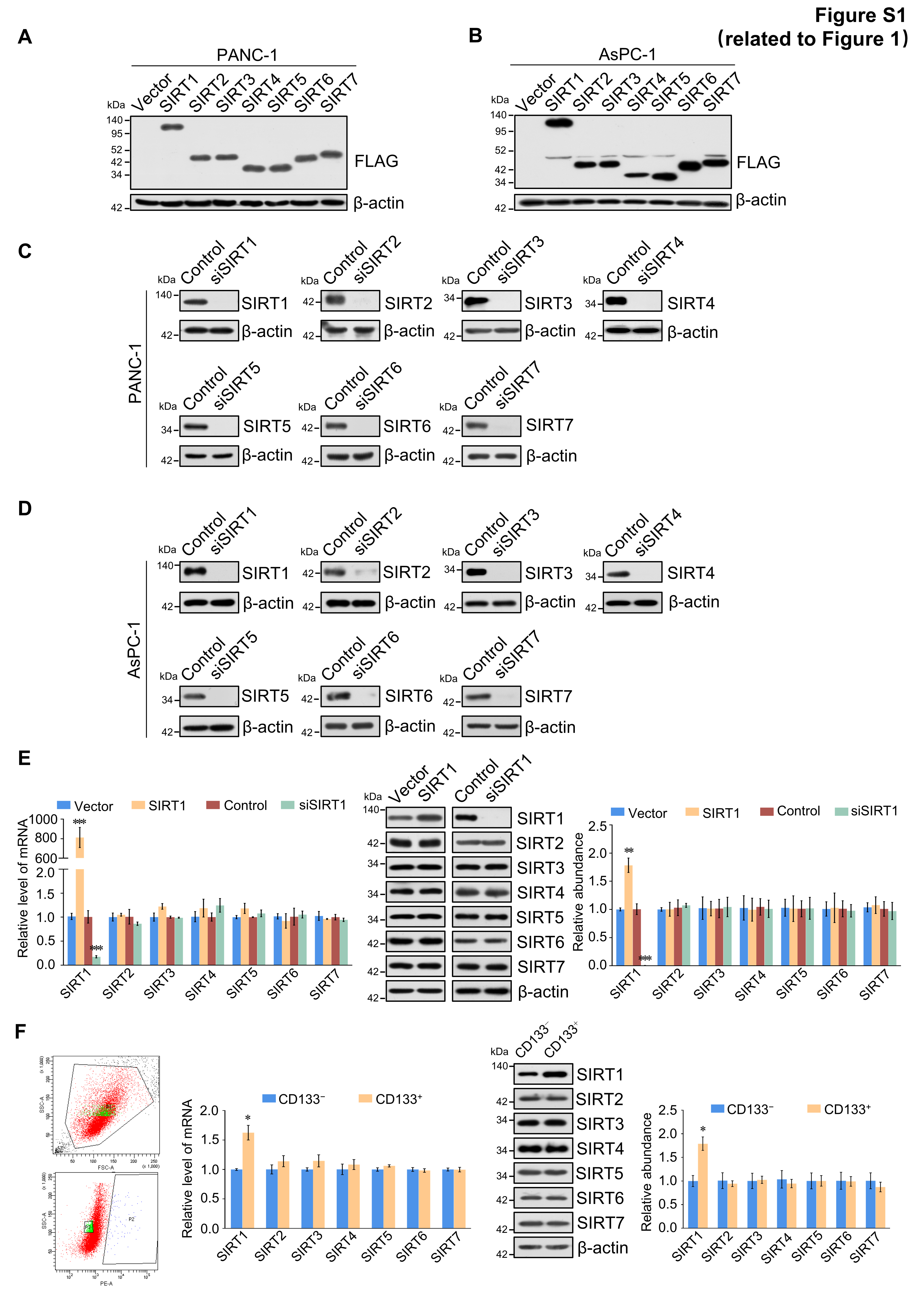

Supplement: Supplementary file 3 — Figure S1 [file 41418_2021_821_MOESM3_ESM.png]

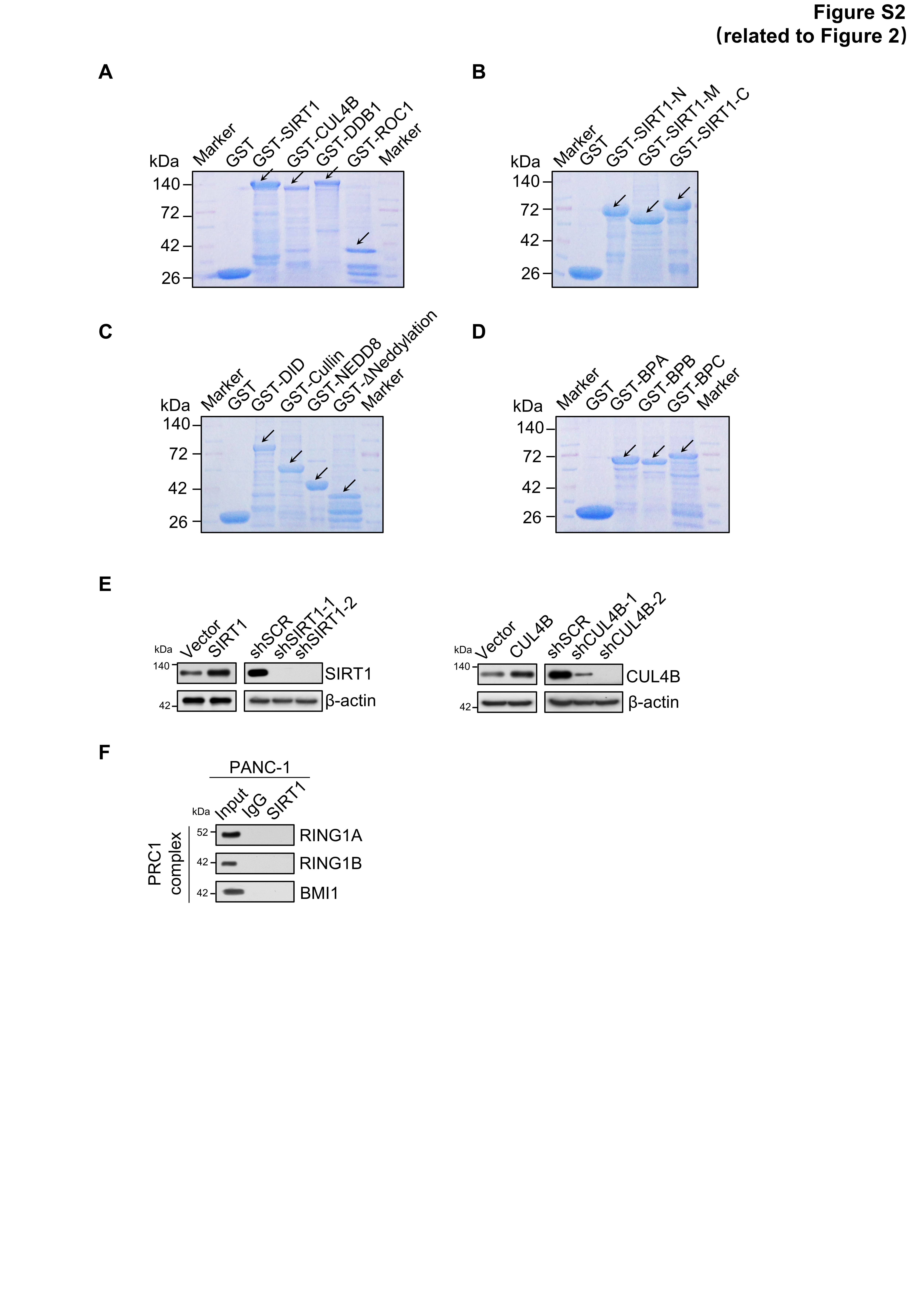

Supplement: Supplementary file 4 — Figure S2 [file 41418_2021_821_MOESM4_ESM.png]

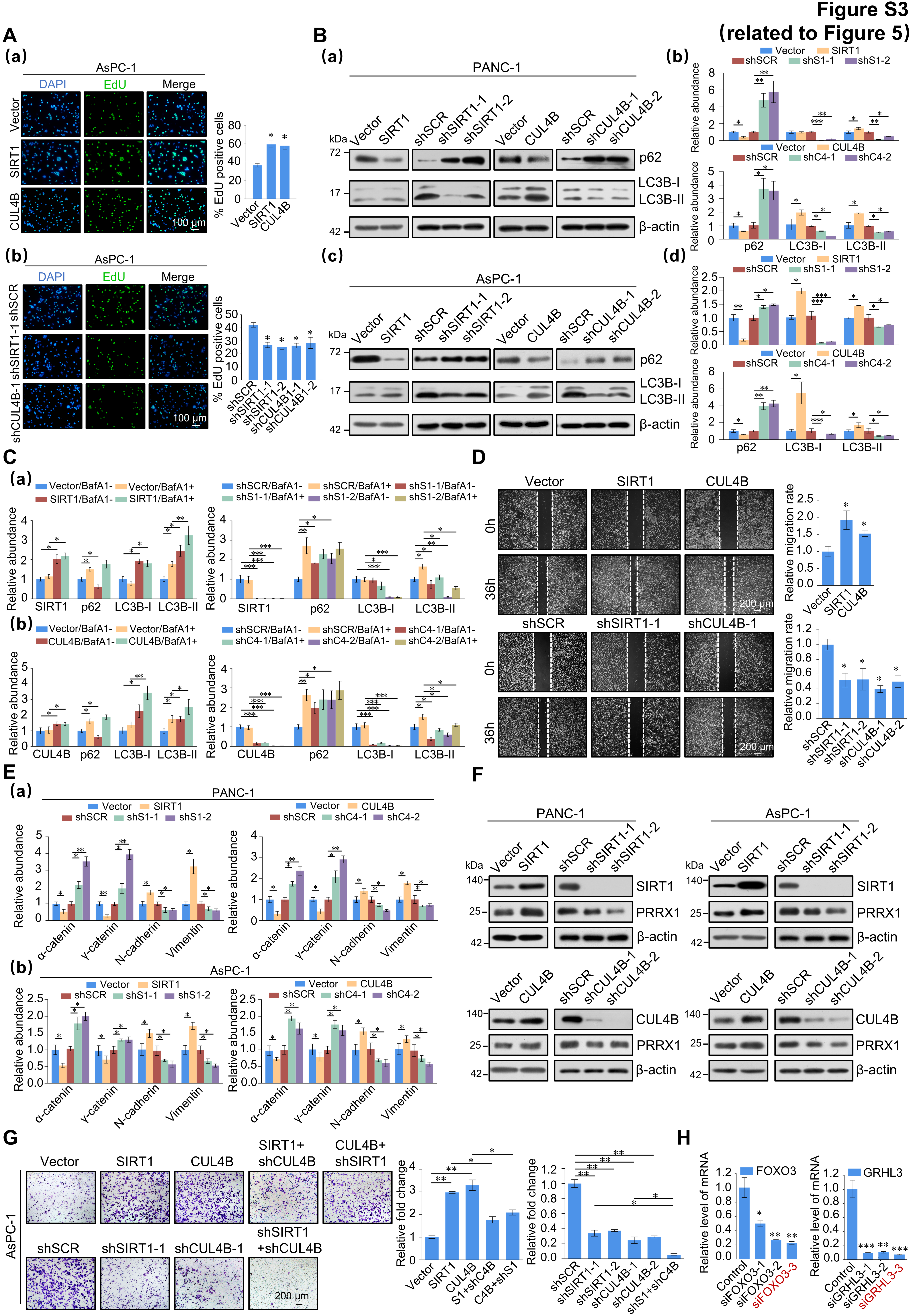

Supplement: Supplementary file 5 — Figure S3 [file 41418_2021_821_MOESM5_ESM.png]

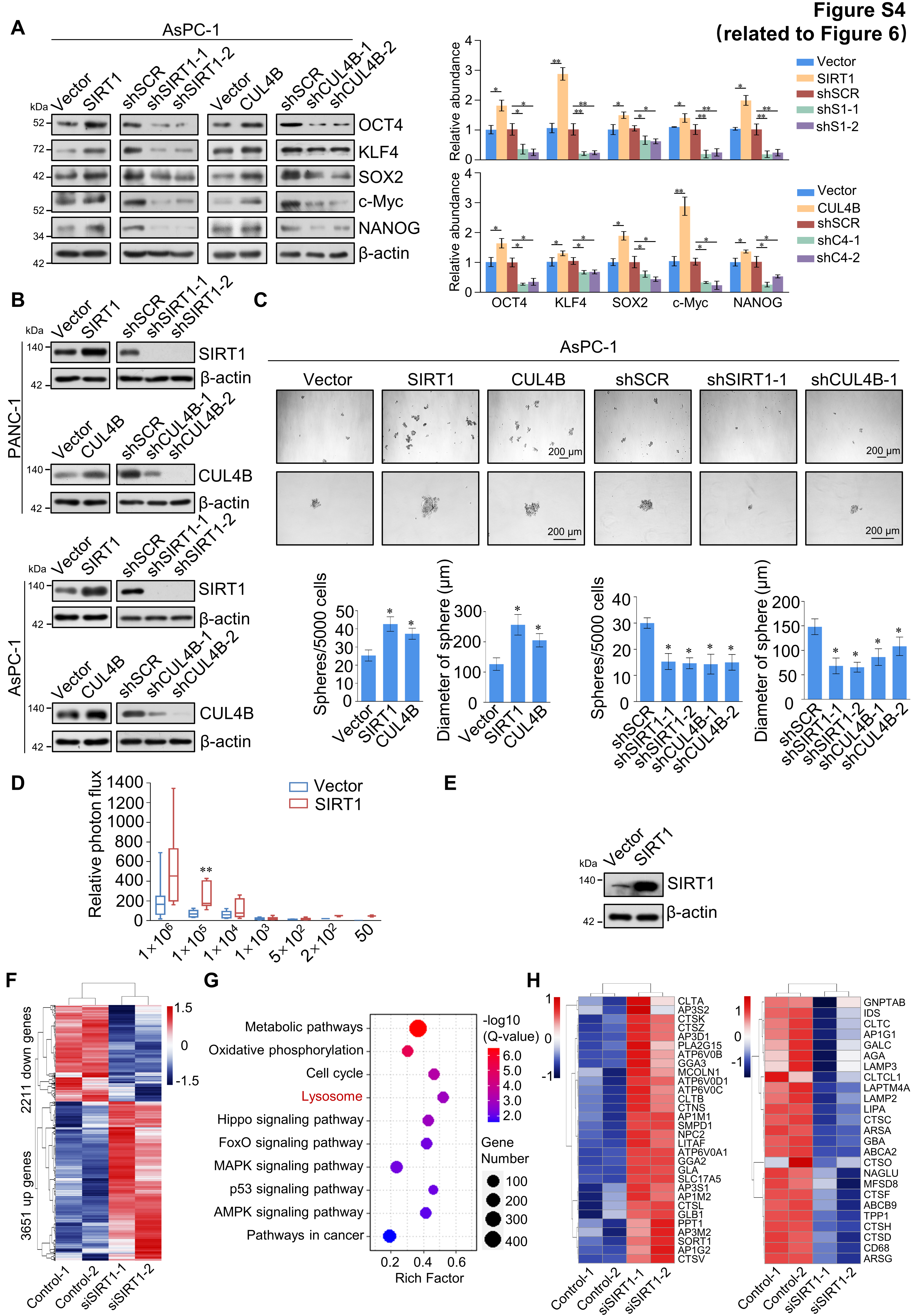

Supplement: Supplementary file 6 — Figure S4 [file 41418_2021_821_MOESM6_ESM.png]

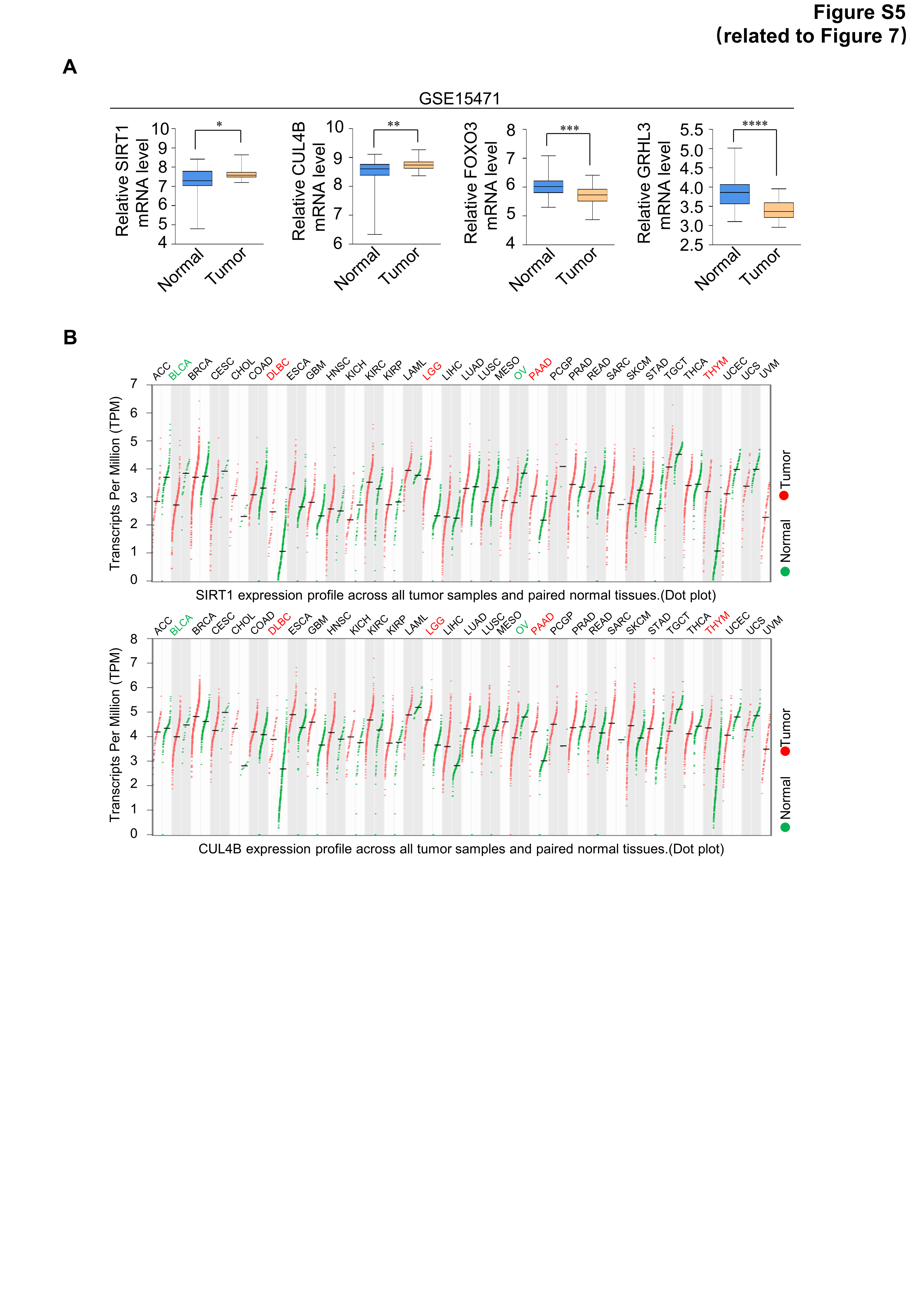

Supplement: Supplementary file 7 — Figure S5 [file 41418_2021_821_MOESM7_ESM.png]
